# Supplementary material for: Roles of GacSA and DJ41_1407 in Acinetobacter baumannii ATCC 19606
Source: Int J Mol Sci. 2025 Oct 31;26(21):10620. doi: 10.3390/ijms262110620 (PMC12609611; doi:10.3390/ijms262110620)
Supplement: Supplementary file 1 [file ijms-26-10620-s001.zip › ijms-3872908-supplementary.pdf]

Roles of GacSA and DJ41_1407 in *Acinetobacter baumannii* ATCC 19606

Supplementary Materials

Supplementary Figures

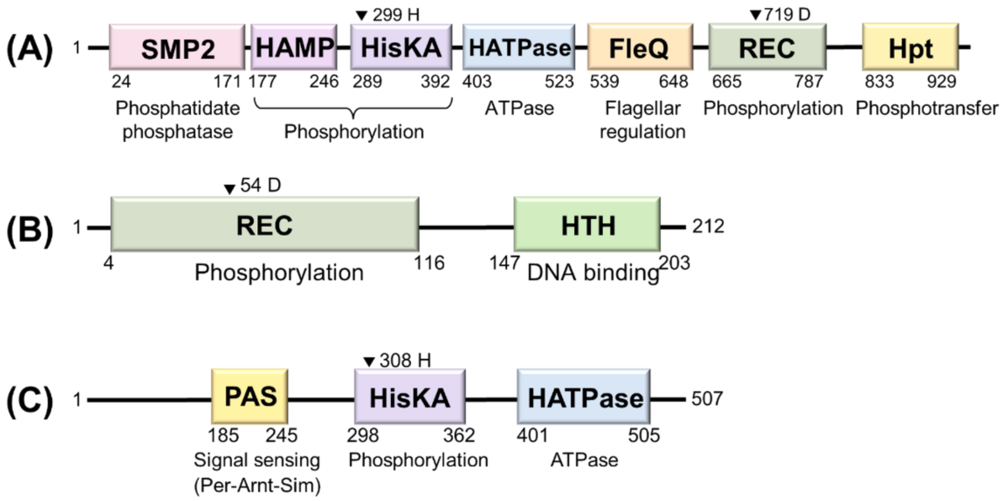

Figure S1. Putative domains of GacS, GacA, and DJ41_1407. Protein conserved domains were analysed using CDvist. (**A**) GacS conserved domains. SMP2: phosphatidate phosphatase domain; HAMP and HisKA: histidine kinase domain for phosphorylation; HATPase: ATPase domain; FleQ: domain for flagellar regulation; REC: receiver domain for phosphorylation; Hpt: histidine-containing phosphotransfer domain. The possible phosphorylation sites are a histidine residue at 299 and an aspartic acid residue at 719. **(B)** GacA conserved domains. REC: receiver domain for phosphorylation; HTH: helix-turn-helix domain for DNA binding. The possible phosphorylation site is the aspartic acid residue at 54. **(C)** DJ41_1407 conserved domains. PAS: Per-Arnt-Sim for signal sensing; HisKA: histidine kinase domain for phosphorylation; HATPase: ATPase domain. The possible phosphorylation site is a histidine residue at 308.

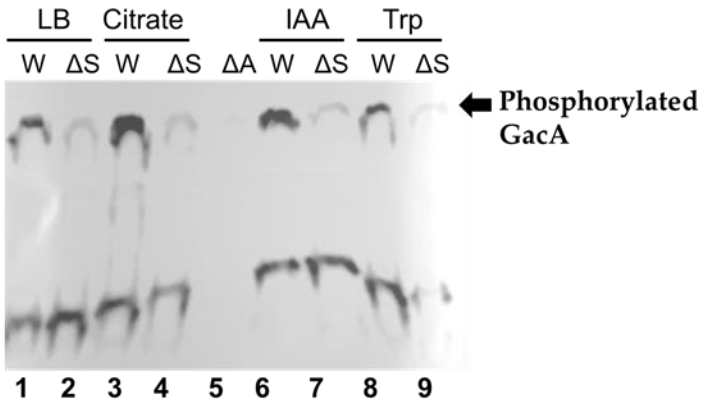

Figure S2. Analysis of the intensity of phosphorylated GacA using Phos-tag™.
W: *A. baumannii* ATCC 19606, ΔS: *A. baumannii* ATCC 19606 Δ*gacS*, ΔA: *A. baumannii* ATCC 19606 Δ*gacA*. LB: strains cultured in LB medium; Citrate: strains cultured in M9 medium with 5 mM citrate; IAA: 5 mM indole-3-acetic acid added; Trp: 5 mM tryptophan added. The black arrow indicates the position of phosphorylated GacA.

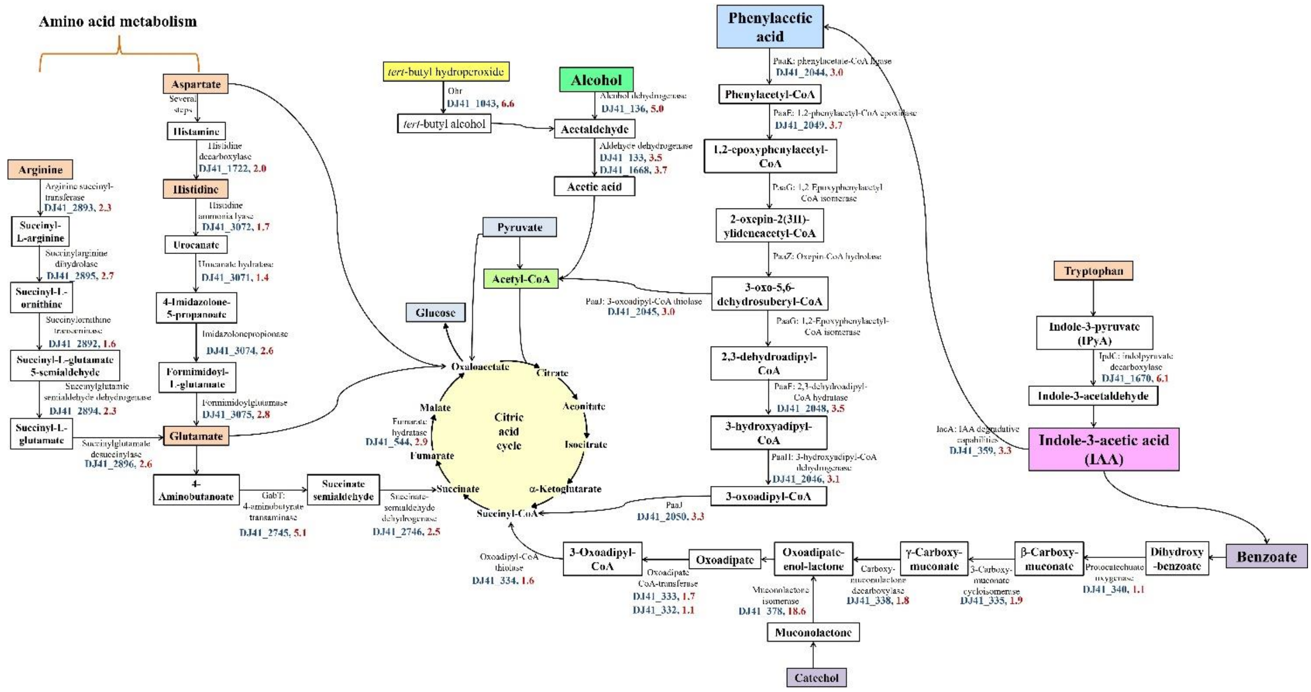

Figure S3. Annotation of genes regulated by GacA in the *A. baumannii* ATCC 19606 carbon metabolism pathway. Blue: gene number; black: gene name; red: log2 change in expression (wild-type/Δ*gacA*). Orange background represents the amino acid metabolic pathway, yellow background represents the organic peroxide metabolic pathway, green background indicates the alcohol metabolic pathway, blue background denotes the PAA metabolic pathway, purple background represents the benzoate metabolic pathway, white background signifies the intermediate compounds produced within the metabolic pathways, and light yellow background represents the citric acid cycle pathway.

Supplementary Tables

Table S1. Differentially regulated genes in the Δ*gacS* mutant strain versus the wild-type strain ATCC 19606 in gene ontology.

Category and locus tag	Gene	Log_2 fold change	p-value	Predicted function
Biological Process (BP)				
transmembrane transport				
DJ41_1671		4.2	1.5E-09	organic Anion Transporter Polypeptide family protein
DJ41_2076		3.6	7.4E-07	sugar and other transporter family protein
DJ41_2413		3.5	3.9E-08	amino acid permease family protein
DJ41_2576		2.3	0.00099	amino acid permease family protein
DJ41_2743		2.2	0.001	major Facilitator Superfamily protein
DJ41_2890		1.7	0.00762	amino acid permease family protein
DJ41_3250		1.6	0.01121	amino ABC transporter permease 3 TM region His Glu Gln Arg opine family domain protein
DJ41_3259		1.5	0.01296	amino acid permease family protein
DJ41_356		1.5	0.04504	sugar and other transporter family protein
DJ41_775		1.5	0.03241	sugar and other transporter family protein
DJ41_776		1.4	0.0226	amino acid permease family protein
DJ41_991		1.4	0.0251	amino ABC transporter permease 3 TM region His Glu Gln Arg opine family domain protein
cell redox homeostasis				
DJ41_568	*lpdA*	2.4	9.1E-05	dihydrolipoyl dehydrogenase
tricarboxylic acid cycle				
DJ41_544		2.3	0.00021	fumarase C family protein
protein folding				
DJ41_2868		2.1	0.00281	proteobacterial lipase chaperone family protein
lipid metabolic process				
DJ41_3715		2.0	0.00189	fatty acid desaturase family protein
Cellular Component (CC)				
integral component of plasma membrane				
DJ41_2076		4.2	1.5E-09	organic Anion Transporter Polypeptide family protein
DJ41_2576		3.6	7.4E-07	sugar and other transporter family protein

DJ41_3250		2.2	0.001	major Facilitator Superfamily protein
DJ41_356		1.5	0.04504	sugar and other transporter family protein
DJ41_991		1.5	0.03241	sugar and other transporter family protein
membrane				
DJ41_1671		3.5	3.9E-08	amino acid permease family protein
DJ41_2413		2.3	0.00099	amino acid permease family protein
DJ41_2743		2.1	0.00281	proteobacterial lipase chaperone family protein
DJ41_2868		1.7	0.00762	amino acid permease family protein
DJ41_2890		1.6	0.01121	amino ABC transporter permease 3 TM region His Glu Gln Arg opine family domain protein
DJ41_3259		1.5	0.01296	amino acid permease family protein
DJ41_775		1.4	0.0226	amino acid permease family protein
DJ41_776		1.4	0.0251	amino ABC transporter permease 3 TM region His Glu Gln Arg opine family domain protein
integral component of membrane				
DJ41_802		2.4	0.00017	dicarboxylate symporter family protein
DJ41_355		2.3	0.0004	outer membrane porin OprD family protein
DJ41_776		1.6	0.01121	amino ABC transporter permease 3 TM region His Glu Gln Arg opine family domain protein
DJ41_775		1.4	0.0251	amino ABC transporter permease 3 TM region His Glu Gln Arg opine family domain protein
DJ41_2978	*rrtA*	1.4	0.03297	rhombosortase
DJ41_343	*oprB*	1.4	0.03304	porin B
DJ41_3022		-1.6	0.0038	sulfite exporter TauE SafE family protein
Molecular Function (MF)				
2 iron, 2 sulfur cluster binding				
DJ41_362		5.4	3.1E-15	ring hydroxylating alpha subunit family protein
flavin adenine dinucleotide binding				
DJ41_359		5.4	5.9E-15	hypothetical protein
DJ41_3253		3.0	3.3E-06	hypothetical protein
iron ion binding				
DJ41_362		5.4	3.1E-15	ring hydroxylating alpha subunit family protein
DJ41_377	*catA*	1.3	0.04319	catechol 1 2 dioxygenase
oxidoreductase activity				
DJ41_362		5.4	3.1E-15	ring hydroxylating alpha subunit family protein
DJ41_365		4.6	1.2E-11	2Fe 2S iron sulfur cluster binding domain protein
DJ41_3716		2.4	7.8E-05	ferric reductase NAD binding domain protein

DJ41_568	*lpdA*	2.4	9.1E-05	dihydrolipoyl dehydrogenase
DJ41_2746		2.0	0.00093	succinate semialdehyde dehydrogenase family protein
DJ41_3256	*mmsA*	2.0	0.00104	methylmalonate semialdehyde dehydrogenase
DJ41_2046		1.7	0.00452	putative 3 hydroxybutyryl CoA dehydrogenase
DJ41_2049		1.6	0.00713	2Fe 2S iron sulfur cluster binding domain protein
DJ41_136		1.6	0.0085	iron containing alcohol dehydrogenase family protein
DJ41_2894	*astD*	1.4	0.02224	succinylglutamic semialdehyde dehydrogenase
DJ41_2414		1.3	0.04465	FAD binding domain protein
DJ41_2409		1.3	0.04097	acyl CoA reductase family protein
DJ41_2254	*benC*	-1.1	0.03702	benzoate 1 2 dioxygenase electron transfer component
DJ41_2750		-1.5	0.00581	pyridine nucleotide disulfide oxidoreductase family protein
oxidoreductase activity, acting on the CH-CH group of donors				
DJ41_359		5.4	5.9E-15	hypothetical protein
DJ41_3253		3.0	3.3E-06	hypothetical protein
DJ41_3245		2.1	0.03884	hypothetical protein
pyridoxal phosphate binding				
DJ41_2745	*gabT*	4.7	1.1E-12	4 aminobutyrate transaminase
DJ41_904	*bioA*	-1.1	0.0378	adenosylmethionine 8 amino 7 oxononanoate transaminase
electron transfer activity				
DJ41_365		4.6	1.2E-11	2Fe 2S iron sulfur cluster binding domain protein
DJ41_2049		1.6	0.00713	2Fe 2S iron sulfur cluster binding domain protein
DJ41_2254	*benC*	-1.1	0.03702	benzoate 1 2 dioxygenase electron transfer component
iron-sulfur cluster binding				
DJ41_365		4.6	1.2E-11	2Fe 2S iron sulfur cluster binding domain protein
DJ41_572	*lipA*	3.7	7.8E-09	lipoyl synthase
DJ41_2049		1.6	0.00713	2Fe 2S iron sulfur cluster binding domain protein
DJ41_741	*bioB*	-1.3	0.01474	biotin synthase
DJ41_2254	*benC*	-1.1	0.03702	benzoate 1 2 dioxygenase electron transfer component
hydrolase activity				
DJ41_372		4.1	1.4E-05	penicillin amidase family protein

DJ41_2209		1.6	0.02106	hypothetical protein
DJ41_3074	*hutI*	1.5	0.01837	imidazolonepropionase
4 iron, 4 sulfur cluster binding				
DJ41_572	*lipA*	3.7	7.8E-09	lipoyl synthase
catalytic activity				
DJ41_572	*lipA*	3.7	7.8E-09	lipoyl synthase
DJ41_3252		3.1	2.5E-06	enoyl CoA hydratase isomerase family protein
DJ41_3254		2.6	2.5E-05	AMP binding enzyme family protein
DJ41_429		2.3	0.01458	3 beta hydroxysteroid dehydrogenase isomerase family protein
DJ41_2044	*paaF*	2.1	0.00059	phenylacetate CoA ligase
DJ41_2047	*paaB*	1.9	0.00178	phenylacetate degradation putative enoyl CoA hydratase PaaB
DJ41_405		1.7	0.01341	AMP binding enzyme family protein
DJ41_907		1.7	0.0063	glycosyltransferase 20 family protein
DJ41_2048		1.5	0.01282	enoyl CoA hydratase isomerase family protein
DJ41_1973		1.5	0.01756	AMP binding enzyme family protein
DJ41_1670		1.4	0.01911	thiamine pyrophosphate enzyme central domain protein
DJ41_377	*catA*	1.3	0.04319	catechol 1 2 dioxygenase
DJ41_906	*otsB*	1.3	0.04392	trehalose phosphatase
DJ41_741	*bioB*	-1.3	0.01474	biotin synthase
FMN binding				
DJ41_366		3.7	1.8E-08	flavin reductase like domain protein
transferase activity				
DJ41_3249		3.7	0.02158	autoinducer synthetase family protein
DNA binding				
DJ41_2209		1.6	0.02106	hypothetical protein
DJ41_1847		1.5	0.01972	arc like DNA binding domain protein
DJ41_2431		1.4	0.02762	hypothetical protein
DJ41_3179	*uvrA*	1.3	0.03682	excinuclease ABC subunit A
DJ41_2430		1.3	0.04232	type I restriction modification DNA specificity domain protein
DJ41_3322		-2.6	5.9E-06	transposase DDE domain protein
acyltransferase activity				
DJ41_569		2.5	4.6E-05	e3 binding domain protein
lyase activity				

DJ41_544		2.3	0.00021	fumarase C family protein
NAD binding				
DJ41_3255	*mmsB*	2.1	0.0008	3 hydroxyisobutyrate dehydrogenase
NADP binding				
DJ41_3255	*mmsB*	2.1	0.0008	3 hydroxyisobutyrate dehydrogenase
unfolded protein binding				
DJ41_2868		2.1	0.00281	proteobacterial lipase chaperone family protein
magnesium ion binding				
DJ41_881	*prs*	2.0	0.00088	ribose phosphate diphosphokinase family protein
DJ41_1670		1.4	0.01911	thiamine pyrophosphate enzyme central domain protein

Table S2. Differentially regulated genes in the Δ*gacS* mutant strain versus the wild-type strain ATCC 19606 in KEGG.

Category and locus tag	Gene	Log_2 fold change	p-value	Predicted function
Two-component system				
DJ41_539		17.9	1.2E-28	response regulator
DJ41_538		3.7	0.02158	autoinducer synthetase family protein
DJ41_775		1.6	0.01121	amino ABC transporter permease 3 TM region His Glu Gln Arg opine family domain protein
DJ41_776		1.4	0.0251	amino ABC transporter permease 3 TM region His Glu Gln Arg opine family domain protein
DJ41_3249		1.3	0.03528	acetyl CoA C acetyltransferase family protein
Alanine, aspartate and glutamate metabolism				
DJ41_2745	*gabT*	4.7	1.1E-12	4 aminobutyrate transaminase
DJ41_544		2.3	0.00021	fumarase C family protein
DJ41_2746		2.0	0.00093	succinate semialdehyde dehydrogenase family protein
Aminobenzoate degradation				
DJ41_358		4.7	1.3E-12	amidase family protein
DJ41_3252		3.1	2.5E-06	enoyl CoA hydratase isomerase family protein
DJ41_2048		1.5	0.01282	enoyl CoA hydratase isomerase family protein
Arginine and proline metabolism				
DJ41_358		4.7	1.3E-12	amidase family protein
DJ41_2895	*astB*	1.6	0.00851	succinylarginine dihydrolase
DJ41_2896	*astE*	1.5	0.01805	succinylglutamate desuccinylase
DJ41_2894	*astD*	1.4	0.02224	succinylglutamic semialdehyde dehydrogenase
DJ41_2414		1.3	0.04465	FAD binding domain protein
beta-Alanine metabolism				
DJ41_2745	*gabT*	4.7	1.1E-12	4 aminobutyrate transaminase
DJ41_3252		3.1	2.5E-06	enoyl CoA hydratase isomerase family protein
DJ41_3253		3.0	3.3E-06	hypothetical protein
DJ41_3256	*mmsA*	2.0	0.00104	methylmalonate semialdehyde dehydrogenase
DJ41_2048		1.5	0.01282	enoyl CoA hydratase isomerase family protein
Biosynthesis of cofactors				

DJ41_364		4.7	4E-12	short chain dehydrogenase family protein
DJ41_572	*lipA*	3.7	7.8E-09	lipoyl synthase
DJ41_568	*lpdA*	2.4	9.1E-05	dihydrolipoyl dehydrogenase
DJ41_904	*bioA*	-1.1	0.0378	adenosylmethionine 8 amino 7 oxononanoate transaminase
DJ41_892	*fabG*	-1.1	0.03022	3 oxoacyl acyl carrier protein reductase
DJ41_2711		-1.1	0.03117	hypothetical protein
DJ41_741	*bioB*	-1.3	0.01474	biotin synthase
DJ41_2710		-1.3	0.01121	hypothetical protein
DJ41_249	*fabZ*	-1.4	0.00637	beta hydroxyacyl acyl carrier protein dehydratase FabZ
Biotin metabolism				
DJ41_364		4.7	4E-12	short chain dehydrogenase family protein
DJ41_904	*bioA*	-1.1	0.0378	adenosylmethionine 8 amino 7 oxononanoate transaminase
DJ41_892	*fabG*	-1.1	0.03022	3 oxoacyl acyl carrier protein reductase
DJ41_741	*bioB*	-1.3	0.01474	biotin synthase
DJ41_2710		-1.3	0.01121	hypothetical protein
DJ41_249	*fabZ*	-1.4	0.00637	beta hydroxyacyl acyl carrier protein dehydratase FabZ
Butanoate metabolism				
DJ41_2745	*gabT*	4.7	1.1E-12	4 aminobutyrate transaminase
DJ41_3252		3.1	2.5E-06	enoyl CoA hydratase isomerase family protein
DJ41_3253		3.0	3.3E-06	hypothetical protein
DJ41_567	*budC*	2.5	4.8E-05	diacetyl reductase S acetoin forming
DJ41_566		2.4	8.7E-05	zinc binding dehydrogenase family protein
DJ41_2746		2.0	0.00093	succinate semialdehyde dehydrogenase family protein
DJ41_2046		1.7	0.00452	putative 3 hydroxybutyryl CoA dehydrogenase
DJ41_2048		1.5	0.01282	enoyl CoA hydratase isomerase family protein
DJ41_539		1.3	0.03528	acetyl CoA C acetyltransferase family protein
Fatty acid biosynthesis				
DJ41_364		4.7	4E-12	short chain dehydrogenase family protein
DJ41_405		1.7	0.01341	AMP binding enzyme family protein
DJ41_892	*fabG*	-1.1	0.03022	3 oxoacyl acyl carrier protein reductase
DJ41_3412	*accD*	-1.1	0.02552	acetyl CoA carboxylase carboxyl transferase beta subunit

DJ41_2710		-1.3	0.01121	hypothetical protein
DJ41_249	*fabZ*	-1.4	0.00637	beta hydroxyacyl acyl carrier protein dehydratase FabZ
DJ41_1052	*accA*	-1.6	0.00247	acetyl CoA carboxylase carboxyl transferase alpha subunit
Fatty acid degradation				
DJ41_364		4.7	4E-12	short chain dehydrogenase family protein
DJ41_3252		3.1	2.5E-06	enoyl CoA hydratase isomerase family protein
DJ41_3253		3.0	3.3E-06	hypothetical protein
DJ41_3253		3.0	3.3E-06	hypothetical protein
DJ41_3716		2.4	7.8E-05	ferric reductase NAD binding domain protein
DJ41_3715		2.0	0.00189	fatty acid desaturase family protein
DJ41_2045	*pcaF*	1.9	0.00179	3 oxoadipyl CoA thiolase
DJ41_2045	*pcaF*	1.9	0.00179	3 oxoadipyl CoA thiolase
DJ41_405		1.7	0.01341	AMP binding enzyme family protein
DJ41_405		1.7	0.01341	AMP binding enzyme family protein
DJ41_136		1.6	0.0085	iron containing alcohol dehydrogenase family protein
DJ41_2048		1.5	0.01282	enoyl CoA hydratase isomerase family protein
DJ41_2048		1.5	0.01282	enoyl CoA hydratase isomerase family protein
DJ41_539		1.3	0.03528	acetyl CoA C acetyltransferase family protein
DJ41_539		1.3	0.03528	acetyl CoA C acetyltransferase family protein
DJ41_892	*fabG*	-1.1	0.03022	3 oxoacyl acyl carrier protein reductase
DJ41_3412	*accD*	-1.1	0.02552	acetyl CoA carboxylase carboxyl transferase beta subunit
DJ41_2710		-1.3	0.01121	hypothetical protein
DJ41_249	*fabZ*	-1.4	0.00637	beta hydroxyacyl acyl carrier protein dehydratase FabZ
DJ41_1052	*accA*	-1.6	0.00247	acetyl CoA carboxylase carboxyl transferase alpha subunit
Phenylalanine metabolism				
DJ41_358		4.7	1.3E-12	amidase family protein
DJ41_2041		3.3	3E-07	phenylacetic acid degradation protein *paaI*
DJ41_2044	*paaF*	2.1	0.00059	phenylacetate CoA ligase
DJ41_2047	*paaB*	1.9	0.00178	phenylacetate degradation putative enoyl CoA hydratase PaaB
DJ41_2046		1.7	0.00452	putative 3 hydroxybutyryl CoA dehydrogenase
DJ41_2049		1.6	0.00713	2Fe 2S iron sulfur cluster binding domain protein

DJ41_2048		1.5	0.01282	enoyl CoA hydratase isomerase family protein
DJ41_2050	*paaJ*	1.5	0.01791	phenylacetate CoA oxygenase PaaJ subunit
Prodigiosin biosynthesis				
DJ41_364		4.7	4E-12	short chain dehydrogenase family protein
DJ41_892	*fabG*	-1.1	0.03022	3 oxoacyl acyl carrier protein reductase
Propanoate metabolism				
DJ41_2745	*gabT*	4.7	1.1E-12	4 aminobutyrate transaminase
DJ41_3252		3.1	2.5E-06	enoyl CoA hydratase isomerase family protein
DJ41_3253		3.0	3.3E-06	hypothetical protein
DJ41_3254		2.6	2.5E-05	AMP binding enzyme family protein
DJ41_568	*lpdA*	2.4	9.1E-05	dihydrolipoyl dehydrogenase
DJ41_3256	*mmsA*	2.0	0.00104	methylmalonate semialdehyde dehydrogenase
DJ41_2048		1.5	0.01282	enoyl CoA hydratase isomerase family protein
DJ41_3412	*accD*	-1.1	0.02552	acetyl CoA carboxylase carboxyl transferase beta subunit
DJ41_1052	*accA*	-1.6	0.00247	acetyl CoA carboxylase carboxyl transferase alpha subunit
Styrene degradation				
DJ41_358		4.7	1.3E-12	amidase family protein
Tryptophan metabolism				
DJ41_358		4.7	1.3E-12	amidase family protein
DJ41_3252		3.1	2.5E-06	enoyl CoA hydratase isomerase family protein
DJ41_568	*lpdA*	2.4	9.1E-05	dihydrolipoyl dehydrogenase
DJ41_2048		1.5	0.01282	enoyl CoA hydratase isomerase family protein
DJ41_1959		1.4	0.02449	catalase family protein
DJ41_539		1.3	0.03528	acetyl CoA C acetyltransferase family protein
Penicillin and cephalosporin biosynthesis				
DJ41_372		4.1	1.4E-05	penicillin amidase family protein
Biofilm formation - Pseudomonas aeruginosa				
DJ41_3249		3.7	0.02158	autoinducer synthetase family protein
Cysteine and methionine metabolism				
DJ41_3249		3.7	0.02158	autoinducer synthetase family protein
Lipoic acid metabolism				
DJ41_572	*lipA*	3.7	7.8E-09	lipoyl synthase
Quorum sensing				
DJ41_3249		3.7	0.02158	autoinducer synthetase family protein
DJ41_405		1.7	0.01341	AMP binding enzyme family protein

Riboflavin metabolism				
DJ41_366		3.7	1.8E-08	flavin reductase like domain protein
Tyrosine metabolism				
DJ41_366		3.7	1.8E-08	flavin reductase like domain protein
DJ41_3252		3.1	2.5E-06	enoyl CoA hydratase isomerase family protein
DJ41_568	*lpdA*	2.4	9.1E-05	dihydrolipoyl dehydrogenase
DJ41_3255	*mmsB*	2.1	0.0008	3 hydroxyisobutyrate dehydrogenase
DJ41_2746		2.0	0.00093	succinate semialdehyde dehydrogenase family protein
DJ41_3256	*mmsA*	2.0	0.00104	methylmalonate semialdehyde dehydrogenase
DJ41_2045	*pcaF*	1.9	0.00179	3 oxoadipyl CoA thiolase
DJ41_136		1.6	0.0085	iron containing alcohol dehydrogenase family protein
DJ41_2048		1.5	0.01282	enoyl CoA hydratase isomerase family protein
DJ41_539		1.3	0.03528	acetyl CoA C acetyltransferase family protein
Benzoate degradation				
DJ41_3252		3.1	2.5E-06	enoyl CoA hydratase isomerase family protein
DJ41_2045	*pcaF*	1.9	0.00179	3 oxoadipyl CoA thiolase
DJ41_2046		1.7	0.00452	putative 3 hydroxybutyryl CoA dehydrogenase
DJ41_2048		1.5	0.01282	enoyl CoA hydratase isomerase family protein
DJ41_539		1.3	0.03528	acetyl CoA C acetyltransferase family protein
DJ41_377	*catA*	1.3	0.04319	catechol 1 2 dioxygenase
DJ41_2253	*benB*	-1.0	0.04776	benzoate 1 2 dioxygenase small subunit
DJ41_2254	*benC*	-1.1	0.03702	benzoate 1 2 dioxygenase electron transfer component
DJ41_2255		-1.1	0.03554	short chain dehydrogenase family protein
Caprolactam degradation				
DJ41_3252		3.1	2.5E-06	enoyl CoA hydratase isomerase family protein
DJ41_2048		1.5	0.01282	enoyl CoA hydratase isomerase family protein
Geraniol degradation				
DJ41_3252		3.1	2.5E-06	enoyl CoA hydratase isomerase family protein
DJ41_2045	*pcaF*	1.9	0.00179	3 oxoadipyl CoA thiolase
DJ41_2048		1.5	0.01282	enoyl CoA hydratase isomerase family protein
Limonene and pinene degradation				
DJ41_3252		3.1	2.5E-06	enoyl CoA hydratase isomerase family protein
Lysine degradation				
DJ41_3252		3.1	2.5E-06	enoyl CoA hydratase isomerase family protein
DJ41_568	*lpdA*	2.4	9.1E-05	dihydrolipoyl dehydrogenase

DJ41_2746		2.0	0.00093	succinate semialdehyde dehydrogenase family protein
DJ41_2048		1.5	0.01282	enoyl CoA hydratase isomerase family protein
DJ41_539		1.3	0.03528	acetyl CoA C acetyltransferase family protein
Carbon metabolism				
DJ41_3253		3.0	3.3E-06	hypothetical protein
DJ41_3254		2.6	2.5E-05	AMP binding enzyme family protein
DJ41_569		2.5	4.6E-05	e3 binding domain protein
DJ41_568	*lpdA*	2.4	9.1E-05	dihydrolipoyl dehydrogenase
DJ41_881	*prs*	2.0	0.00088	ribose phosphate diphosphokinase family protein
DJ41_3256	*mmsA*	2.0	0.00104	methylmalonate semialdehyde dehydrogenase
DJ41_2046		1.7	0.00452	putative 3 hydroxybutyryl CoA dehydrogenase
DJ41_1959		1.4	0.02449	catalase family protein
DJ41_2528		1.3	0.03195	isocitrate lyase
DJ41_539		1.3	0.03528	acetyl CoA C acetyltransferase family protein
DJ41_3412	*accD*	-1.1	0.02552	acetyl CoA carboxylase carboxyl transferase beta subunit
DJ41_1052	*accA*	-1.6	0.00247	acetyl CoA carboxylase carboxyl transferase alpha subunit
Carbon fixation pathways in prokaryotes				
DJ41_3254		2.6	2.5E-05	AMP binding enzyme family protein
DJ41_539		1.3	0.03528	acetyl CoA C acetyltransferase family protein
DJ41_3412	*accD*	-1.1	0.02552	acetyl CoA carboxylase carboxyl transferase beta subunit
DJ41_1052	*accA*	-1.6	0.00247	acetyl CoA carboxylase carboxyl transferase alpha subunit
Glycolysis / Gluconeogenesis				
DJ41_3254		2.6	2.5E-05	AMP binding enzyme family protein
DJ41_569		2.5	4.6E-05	e3 binding domain protein
DJ41_568	*lpdA*	2.4	9.1E-05	dihydrolipoyl dehydrogenase
Nucleotide metabolism				
DJ41_2077		2.6	0.00011	cytidine and deoxycytidylate deaminase zinc binding region family protein
DJ41_1568		2.4	0.00013	putative deoxyguanosinetriphosphate triphosphohydrolase
Purine metabolism				

DJ41_2077		2.6	0.00011	cytidine and deoxycytidylate deaminase zinc binding region family protein
DJ41_1568		2.4	0.00013	putative deoxyguanosinetriphosphate triphosphohydrolase
DJ41_881	*prs*	2.0	0.00088	ribose phosphate diphosphokinase family protein
Pyruvate metabolism				
DJ41_3254		2.6	2.5E-05	AMP binding enzyme family protein
DJ41_569		2.5	4.6E-05	e3 binding domain protein
DJ41_568	*lpdA*	2.4	9.1E-05	dihydrolipoyl dehydrogenase
DJ41_136		1.6	0.0085	iron containing alcohol dehydrogenase family protein
DJ41_539		1.3	0.03528	acetyl CoA C acetyltransferase family protein
DJ41_3412	*accD*	-1.1	0.02552	acetyl CoA carboxylase carboxyl transferase beta subunit
DJ41_1052	*accA*	-1.6	0.00247	acetyl CoA carboxylase carboxyl transferase alpha subunit
Citrate cycle (TCA cycle)				
DJ41_569		2.5	4.6E-05	e3 binding domain protein
DJ41_568	*lpdA*	2.4	9.1E-05	dihydrolipoyl dehydrogenase
Biosynthesis of unsaturated fatty acids				
DJ41_3716		2.4	7.8E-05	ferric reductase NAD binding domain protein
DJ41_3715		2.0	0.00189	fatty acid desaturase family protein
Glyoxylate and dicarboxylate metabolism				
DJ41_568	*lpdA*	2.4	9.1E-05	dihydrolipoyl dehydrogenase
DJ41_1959		1.4	0.02449	catalase family protein
DJ41_2528		1.3	0.03195	isocitrate lyase
DJ41_539		1.3	0.03528	acetyl CoA C acetyltransferase family protein
DJ41_3074	*hutI*	1.5	0.01837	imidazolonepropionase
DJ41_253	*recA*	1.7	0.00531	protein RecA
Biofilm formation - Vibrio cholerae				
DJ41_2044	*paaF*	2.1	0.00059	phenylacetate CoA ligase
Biosynthesis of amino acids				
DJ41_881	*prs*	2.0	0.00088	ribose phosphate diphosphokinase family protein
Inositol phosphate metabolism				
DJ41_3256	*mmsA*	2.0	0.00104	methylmalonate semialdehyde dehydrogenase
Nicotinate and nicotinamide metabolism				
DJ41_2746		2.0	0.00093	succinate semialdehyde dehydrogenase family protein

Pentose phosphate pathway				
DJ41_881	*prs*	2.0	0.00088	ribose phosphate diphosphokinase family protein

Table S3. Differentially regulated genes in the Δ*gacA* mutant strain versus the wild-type strain ATCC 19606 in gene ontology.

Category and locus tag	Gene	Log_2 fold change	p-value	Predicted function
Biological Process (BP)				
phosphorelay signal transduction system				
DJ41_1406		10.7	2.1E-30	bacterial regulatory s *luxR* family protein
DJ41_873		-1.4	0.03256	his Kinase A domain protein
regulation of transcription, DNA-templated				
DJ41_1406		10.7	2.1E-30	bacterial regulatory s *luxR* family protein
DJ41_2411		2.2	0.00037	bacterial regulatory s *gntR* family protein
DJ41_367		2.0	0.00237	helix turn helix domain protein
DJ41_527		1.9	0.00194	helix turn helix domain protein
DJ41_3247		1.5	0.04132	bacterial regulatory s *luxR* family protein
transmembrane transport				
DJ41_356		5.6	2.8E-13	organic Anion Transporter Polypeptide family protein
DJ41_1671		5.2	1.4E-13	amino acid permease family protein
DJ41_2743		4.1	1.5E-09	amino acid permease family protein
DJ41_3250		3.8	1.4E-07	sugar and other transporter family protein
DJ41_337		3.6	1.2E-07	MFS transporter aromatic acid H symporter family protein
DJ41_132	*eat*	3.5	4.1E-07	ethanolamine permease
DJ41_546		3.3	1.1E-06	sugar and other transporter family protein
DJ41_1746		2.7	2.1E-05	amino acid peptide transporter family protein
DJ41_2076		2.7	4.9E-05	major Facilitator Superfamily protein
DJ41_3174		2.7	2.1E-05	transporter solute sodium symporter family protein
DJ41_3065	*aroP*	2.5	6.2E-05	aromatic amino acid transport protein AroP
DJ41_2377		2.4	0.00674	sugar and other transporter family protein
DJ41_776		2.2	0.00034	amino ABC transporter permease 3 TM region His Glu Gln Arg opine family domain protein
DJ41_2413		2.1	0.00151	amino acid permease family protein
DJ41_2576		2.0	0.00265	sugar and other transporter family protein
DJ41_2584		1.9	0.00375	sugar and other transporter family protein

DJ41_775		1.9	0.00206	amino ABC transporter permease 3 TM region His Glu Gln Arg opine family domain protein
DJ41_2890		1.6	0.00787	amino acid permease family protein
DJ41_2135		1.4	0.02096	sugar and other transporter family protein
DJ41_991		1.4	0.03458	sugar and other transporter family protein
DJ41_3073		1.4	0.02259	amino acid permease family protein
DJ41_262		1.3	0.0449	amino acid permease family protein
DJ41_2531	*citN*	1.3	0.02724	citrate transporter
DJ41_825		-1.5	0.02645	binding dependent transport system inner membrane component family protein
DJ41_827	*tauA*	-1.6	0.01313	taurine ABC transporter periplasmic binding protein
cell redox homeostasis				
DJ41_568	*lpdA*	4.6	2.7E-11	dihydrolipoyl dehydrogenase
tricarboxylic acid cycle				
DJ41_544		2.8	7.2E-06	fumarase C family protein
DJ41_106	*acnB*	-1.4	0.01834	aconitate hydratase 2
signal transduction				
DJ41_1407		-2.6	6.4E-05	PAS fold family protein
DJ41_873		-1.4	0.03256	his Kinase A domain protein
amino acid transport				
DJ41_776		2.2	0.00034	amino ABC transporter permease 3 TM region His Glu Gln Arg opine family domain protein
DJ41_775		1.9	0.00206	amino ABC transporter permease 3 TM region His Glu Gln Arg opine family domain protein
protein folding				
DJ41_2868		2.0	0.00253	proteobacterial lipase chaperone family protein
response to oxidative stress				
DJ41_1959		2.0	0.00122	catalase family protein
Cellular Component (CC)				
membrane				
DJ41_559		8.5	2.2E-24	hypothetical protein
DJ41_1671		5.2	1.4E-13	amino acid permease family protein
DJ41_2743		4.1	1.5E-09	amino acid permease family protein
DJ41_132	*eat*	3.5	4.1E-07	ethanolamine permease
DJ41_1746		2.7	2.1E-05	amino acid peptide transporter family protein

DJ41_3174		2.7	2.1E-05	transporter solute sodium symporter family protein
DJ41_3065	*aroP*	2.5	6.2E-05	aromatic amino acid transport protein AroP
DJ41_776		2.2	0.00034	amino ABC transporter permease 3 TM region His Glu Gln Arg opine family domain protein
DJ41_2413		2.1	0.00151	amino acid permease family protein
DJ41_2868		2.0	0.00253	proteobacterial lipase chaperone family protein
DJ41_406		1.9	0.00576	sodium Bile acid symporter family protein
DJ41_775		1.9	0.00206	amino ABC transporter permease 3 TM region His Glu Gln Arg opine family domain protein
DJ41_2890		1.6	0.00787	amino acid permease family protein
DJ41_3073		1.4	0.02259	amino acid permease family protein
DJ41_262		1.3	0.0449	amino acid permease family protein
DJ41_529		-1.4	0.02416	citrate transporter family protein
DJ41_825		-1.5	0.02645	binding dependent transport system inner membrane component family protein
DJ41_296	*cydB*	-1.7	0.00501	cytochrome d ubiquinol oxidase subunit II
DJ41_2903		-5.1	5.1E-13	*eamA* like transporter family protein
integral component of plasma membrane				
DJ41_2076		5.6	2.8E-13	organic Anion Transporter Polypeptide family protein
DJ41_2135		3.8	1.4E-07	sugar and other transporter family protein
DJ41_2377		3.6	1.2E-07	MFS transporter aromatic acid H symporter family protein
DJ41_2576		3.3	1.1E-06	sugar and other transporter family protein
DJ41_2584		2.7	4.9E-05	major Facilitator Superfamily protein
DJ41_3250		2.4	0.00674	sugar and other transporter family protein
DJ41_337		2.0	0.00265	sugar and other transporter family protein
DJ41_356		1.9	0.00375	sugar and other transporter family protein
DJ41_546		1.4	0.02096	sugar and other transporter family protein
DJ41_991		1.4	0.03458	sugar and other transporter family protein
integral component of membrane				
DJ41_95	*kdpB*	3.7	1.9E-08	K transporting ATPase B subunit
DJ41_96	*kdpC*	3.5	4.7E-07	K transporting ATPase C subunit
DJ41_546		3.3	1.1E-06	sugar and other transporter family protein
DJ41_343	*oprB*	2.8	1.9E-05	porin B
DJ41_355		2.3	0.00029	outer membrane porin OprD family protein

DJ41_776		2.2	0.00034	amino ABC transporter permease 3 TM region His Glu Gln Arg opine family domain protein
DJ41_775		1.9	0.00206	amino ABC transporter permease 3 TM region His Glu Gln Arg opine family domain protein
DJ41_344	*quiA*	1.9	0.00262	quinate shikimate dehydrogenase
DJ41_3022		1.7	0.01097	sulfite exporter TauE SafE family protein
DJ41_802		1.3	0.02825	dicarboxylate symporter family protein
DJ41_2531	*citN*	1.3	0.02724	citrate transporter
DJ41_2978	*rrtA*	1.3	0.03316	rhombosortase
DJ41_1428		1.2	0.04363	outer membrane porin OprD family protein
DJ41_2903		-5.1	5.1E-13	*eamA* like transporter family protein
outer membrane				
DJ41_1773		2.8	9.8E-06	*ompW* family protein
Molecular Function (MF)				
flavin adenine dinucleotide binding				
DJ41_2001		8.5	2.2E-24	hypothetical protein
DJ41_2913		4.2	1.4E-09	hypothetical protein
DJ41_3253		2.0	0.00537	hypothetical protein
DJ41_3395		-1.3	0.04005	hypothetical protein
DJ41_359		-1.3	0.03296	hypothetical protein
oxidoreductase activity, acting on the CH-CH group of donors				
DJ41_2001		8.5	2.2E-24	hypothetical protein
DJ41_2913		4.2	1.4E-09	hypothetical protein
DJ41_3245		2.0	0.00537	hypothetical protein
DJ41_3253		1.9	0.03117	hypothetical protein
DJ41_3395		-1.3	0.04005	hypothetical protein
DJ41_359		-1.3	0.03296	hypothetical protein
oxidoreductase activity				
DJ41_362		6.4	1.4E-17	ring hydroxylating alpha subunit family protein
DJ41_3256	*mmsA*	5.4	2.7E-14	methylmalonate semialdehyde dehydrogenase
DJ41_365		4.9	3.1E-12	2Fe 2S iron sulfur cluster binding domain protein
DJ41_568	*lpdA*	4.6	2.7E-11	dihydrolipoyl dehydrogenase
DJ41_136		4.3	2.3E-10	iron containing alcohol dehydrogenase family protein
DJ41_1668		3.3	2.3E-07	aldehyde dehydrogenase family protein
DJ41_2409		3.2	6.2E-07	acyl CoA reductase family protein
DJ41_133		3.0	2.5E-06	aldehyde dehydrogenase family protein

DJ41_2046		2.9	5.1E-06	putative 3 hydroxybutyryl CoA dehydrogenase
DJ41_2049		2.3	0.00014	2Fe 2S iron sulfur cluster binding domain protein
DJ41_2590		2.2	0.00443	rieske 2Fe 2S domain protein
DJ41_2131		2.2	0.00054	3 hydroxyacyl CoA dehydrogenase NAD binding domain protein
DJ41_2746		2.1	0.00057	succinate semialdehyde dehydrogenase family protein
DJ41_2585		1.9	0.00565	NAD P binding family protein
DJ41_2378		1.9	0.00905	rieske 2Fe 2S domain protein
DJ41_2579		1.8	0.00592	aldehyde dehydrogenase family protein
DJ41_1993		1.8	0.02469	3 hydroxyacyl CoA dehydrogenase NAD binding domain protein
DJ41_2580		1.6	0.01903	homoserine dehydrogenase NAD binding domain protein
DJ41_2588		1.4	0.04109	rieske 2Fe 2S domain protein
DJ41_2894	*astD*	1.3	0.02664	succinylglutamic semialdehyde dehydrogenase
DJ41_2718	*gabD*	1.3	0.0364	succinate semialdehyde dehydrogenase
DJ41_3613		-1.3	0.03905	flavin oxidoreductase NADH oxidase family protein
2 iron, 2 sulfur cluster binding				
DJ41_2378		6.4	1.4E-17	ring hydroxylating alpha subunit family protein
DJ41_2588		2.2	0.00443	rieske 2Fe 2S domain protein
DJ41_2590		1.9	0.00905	rieske 2Fe 2S domain protein
DJ41_362		1.4	0.04109	rieske 2Fe 2S domain protein
iron ion binding				
DJ41_362		6.4	1.4E-17	ring hydroxylating alpha subunit family protein
catalytic activity				
DJ41_572	*lipA*	6.3	5.3E-17	lipoyl synthase
DJ41_3254		5.1	7E-13	AMP binding enzyme family protein
DJ41_1670		5.0	8.6E-13	thiamine pyrophosphate enzyme central domain protein
DJ41_906	*otsB*	3.8	2.1E-08	trehalose phosphatase
DJ41_3252		3.6	1E-07	enoyl CoA hydratase isomerase family protein
DJ41_907		3.2	4.5E-07	glycosyltransferase 20 family protein
DJ41_2044	*paaF*	3.1	1.4E-06	phenylacetate CoA ligase
DJ41_340	*pcaG*	3.0	1.3E-05	protocatechuate 3 4 dioxygenase alpha subunit
DJ41_339	*pcaH*	2.9	1.7E-05	protocatechuate 3 4 dioxygenase beta subunit

DJ41_2047	*paaB*	2.9	5.6E-06	phenylacetate degradation putative enoyl CoA hydratase PaaB
DJ41_2048		2.7	1.8E-05	enoyl CoA hydratase isomerase family protein
DJ41_1070		2.5	7.8E-05	AMP binding enzyme family protein
DJ41_87		2.0	0.00097	AMP binding enzyme family protein
DJ41_1973		1.8	0.00256	AMP binding enzyme family protein
DJ41_2578		1.8	0.00739	thiamine pyrophosphate enzyme central domain protein
DJ41_405		1.8	0.00765	AMP binding enzyme family protein
DJ41_3246		1.7	0.02546	AMP binding enzyme family protein
DJ41_429		1.6	0.04451	3 beta hydroxysteroid dehydrogenase isomerase family protein
DJ41_3165	*acs*	1.5	0.01024	acetate CoA ligase
DJ41_1247		-1.3	0.04616	AMP binding enzyme family protein
4 iron, 4 sulfur cluster binding				
DJ41_572	*lipA*	6.3	5.3E-17	lipoyl synthase
DJ41_106	*acnB*	-1.4	0.01834	aconitate hydratase 2
iron-sulfur cluster binding				
DJ41_572	*lipA*	6.3	5.3E-17	lipoyl synthase
DJ41_365		4.9	3.1E-12	2Fe 2S iron sulfur cluster binding domain protein
DJ41_2049		2.3	0.00014	2Fe 2S iron sulfur cluster binding domain protein
NAD binding				
DJ41_3255	*mmsB*	5.5	2.6E-14	3 hydroxyisobutyrate dehydrogenase
NADP binding				
DJ41_3255	*mmsB*	5.5	2.6E-14	3 hydroxyisobutyrate dehydrogenase
DJ41_2580		1.6	0.01903	homoserine dehydrogenase NAD binding domain protein
magnesium ion binding				
DJ41_1670		5.0	8.6E-13	thiamine pyrophosphate enzyme central domain protein
DJ41_442		2.0	0.00138	phosphoribosyl transferase domain protein
DJ41_2578		1.8	0.00739	thiamine pyrophosphate enzyme central domain protein
DJ41_881	*prs*	1.3	0.02943	ribose phosphate diphosphokinase family protein
pyridoxal phosphate binding				
DJ41_2745	*gabT*	5.0	6.6E-13	4 aminobutyrate transaminase
electron transfer activity				

DJ41_365		4.9	3.1E-12	2Fe 2S iron sulfur cluster binding domain protein
DJ41_2049		2.3	0.00014	2Fe 2S iron sulfur cluster binding domain protein
DJ41_297	*cydA*	-1.7	0.00685	cytochrome d ubiquinol oxidase subunit 1
acyltransferase activity				
DJ41_569		4.8	3.4E-12	e3 binding domain protein
metal ion binding				
DJ41_136		4.3	2.3E-10	iron containing alcohol dehydrogenase family protein
DJ41_3075	*hutG*	1.7	0.00781	formimidoylglutamase
FMN binding				
DJ41_366		4.2	6.3E-10	flavin reductase like domain protein
DJ41_3613		-1.3	0.03905	flavin oxidoreductase NADH oxidase family protein
ATP binding				
DJ41_95	*kdpB*	3.7	1.9E-08	K transporting ATPase B subunit
DJ41_487		1.4	0.04048	miro like family protein
DJ41_253	*recA*	1.3	0.02254	protein RecA
DJ41_3179	*uvrA*	1.3	0.02438	excinuclease ABC subunit A
DJ41_774		1.3	0.03199	ABC transporter family protein
transmembrane transporter activity				
DJ41_132	*eat*	3.5	4.1E-07	ethanolamine permease
DJ41_546		3.3	1.1E-06	sugar and other transporter family protein
DJ41_1746		2.7	2.1E-05	amino acid peptide transporter family protein
DJ41_3174		2.7	2.1E-05	transporter solute sodium symporter family protein
DJ41_827	*tauA*	-1.6	0.01313	taurine ABC transporter periplasmic binding protein
CoA-transferase activity				
DJ41_332	*catI*	3.4	5.1E-07	3 oxoadipate CoA transferase subunit A
DJ41_333	*catJ*	3.2	1.2E-06	3 oxoadipate CoA transferase subunit B
DJ41_2000		1.8	0.00516	coA transferase III family protein
DJ41_375	*catJ*	1.4	0.03146	3 oxoadipate CoA transferase subunit B
acyltransferase activity, transferring groups other than amino-acyl groups				
DJ41_334	*pcaF*	3.2	1E-06	3 oxoadipyl CoA thiolase
DJ41_2045	*pcaF*	3.1	1.2E-06	3 oxoadipyl CoA thiolase
DJ41_179		-2.1	0.00061	acetyl CoA C acetyltransferase family protein
hydrolase activity				

DJ41_372		3.2	0.00011	penicillin amidase family protein
DJ41_371		2.0	0.02353	penicillin amidase family protein
DJ41_3074	*hutI*	1.5	0.01125	imidazolonepropionase
DJ41_1745		1.3	0.03482	metallopeptidase M24 family protein
DJ41_2522	*ureC*	-1.4	0.02276	urease alpha subunit
lyase activity				
DJ41_544		2.8	7.2E-06	fumarase C family protein
transferase activity				
DJ41_3249		2.8	0.02373	autoinducer synthetase family protein
DJ41_846	*mdcA*	1.3	0.03612	malonate decarboxylase alpha subunit
phosphorelay sensor kinase activity				
DJ41_1407		-2.6	6.4E-05	PAS fold family protein
DJ41_873		-1.4	0.03256	his Kinase A domain protein
DNA-binding transcription factor activity				
DJ41_2411		2.2	0.00037	bacterial regulatory s *gntR* family protein
DJ41_367		2.0	0.00237	helix turn helix domain protein
DJ41_527		1.9	0.00194	helix turn helix domain protein
heme binding				
DJ41_1959		2.0	0.00122	catalase family protein
sequence-specific DNA binding				
DJ41_367		2.0	0.00237	helix turn helix domain protein
DJ41_527		1.9	0.00194	helix turn helix domain protein
unfolded protein binding				
DJ41_2868		2.0	0.00253	proteobacterial lipase chaperone family protein

Table S4. Differentially regulated genes in the Δ*gacA* mutant strain versus the wild-type strain ATCC 19606 in KEGG.

Category and locus tag	Gene	Log_2 fold change	p-value	Predicted function
Biofilm formation - Escherichia coli				
DJ41_1406		10.7	2.1E-30	bacterial regulatory s *luxR* family protein
Biofilm formation - Pseudomonas aeruginosa				
DJ41_1406		10.7	2.1E-30	bacterial regulatory s *luxR* family protein
DJ41_3249		2.8	0.02373	autoinducer synthetase family protein
Biofilm formation - Vibrio cholerae				
DJ41_1406		10.7	2.1E-30	bacterial regulatory s *luxR* family protein
DJ41_2044	*paaF*	3.1	1.4E-06	phenylacetate CoA ligase
Two-component system				
DJ41_1406		10.7	2.1E-30	bacterial regulatory s *luxR* family protein
DJ41_94	*kdpA*	4.0	7.1E-09	K transporting ATPase A subunit
DJ41_95	*kdpB*	3.7	1.9E-08	K transporting ATPase B subunit
DJ41_96	*kdpC*	3.5	4.7E-07	K transporting ATPase C subunit
DJ41_3249		2.8	0.02373	autoinducer synthetase family protein
DJ41_776		2.2	0.00034	amino ABC transporter permease 3 TM region His Glu Gln Arg opine family domain protein
DJ41_777		2.2	0.0004	bacterial extracellular solute binding s 3 family protein
DJ41_775		1.9	0.00206	amino ABC transporter permease 3 TM region His Glu Gln Arg opine family domain protein
DJ41_774		1.3	0.03199	ABC transporter family protein
DJ41_538		1.2	0.0465	C4 dicarboxylate anaerobic carrier family protein
DJ41_297	*cydA*	-1.7	0.00685	cytochrome d ubiquinol oxidase subunit 1
DJ41_295		-1.7	0.00679	cyd operon protein YbgT
DJ41_296	*cydB*	-1.7	0.00501	cytochrome d ubiquinol oxidase subunit II
DJ41_179		-2.1	0.00061	acetyl CoA C acetyltransferase family protein
DJ41_803	*ansB*	-2.3	0.0002	glutaminase asparaginase
DJ41_1407		-2.6	6.4E-05	PAS fold family protein
Aminobenzoate degradation				
DJ41_358		7.4	4.9E-21	amidase family protein
DJ41_3252		3.6	1E-07	enoyl CoA hydratase isomerase family protein

DJ41_2048		2.7	1.8E-05	enoyl CoA hydratase isomerase family protein
DJ41_2378		1.9	0.00905	rieske 2Fe 2S domain protein
Arginine and proline metabolism				
DJ41_358		7.4	4.9E-21	amidase family protein
DJ41_2895	*astB*	1.6	0.00871	succinylarginine dihydrolase
DJ41_2896	*astE*	1.5	0.01054	succinylglutamate desuccinylase
DJ41_2893	*astA*	1.5	0.01108	arginine N succinyltransferase
DJ41_2231		1.4	0.01821	pyrroline 5 carboxylate reductase dimerization family protein
DJ41_2894	*astD*	1.3	0.02664	succinylglutamic semialdehyde dehydrogenase
Phenylalanine metabolism				
DJ41_358		7.4	4.9E-21	amidase family protein
DJ41_2041		4.0	4.2E-09	phenylacetic acid degradation protein *paaI*
DJ41_3252		3.6	1E-07	enoyl CoA hydratase isomerase family protein
DJ41_2044	*paaF*	3.1	1.4E-06	phenylacetate CoA ligase
DJ41_2046		2.9	5.1E-06	putative 3 hydroxybutyryl CoA dehydrogenase
DJ41_2047	*paaB*	2.9	5.6E-06	phenylacetate degradation putative enoyl CoA hydratase PaaB
DJ41_2048		2.7	1.8E-05	enoyl CoA hydratase isomerase family protein
DJ41_2049		2.3	0.00014	2Fe 2S iron sulfur cluster binding domain protein
DJ41_2050	*paaJ*	2.2	0.00038	phenylacetate CoA oxygenase PaaJ subunit
DJ41_2585		1.9	0.00565	NAD P binding family protein
DJ41_2051	*paaC*	1.9	0.00149	phenylacetic acid degradation protein paaC
DJ41_1993		1.8	0.02469	3 hydroxyacyl CoA dehydrogenase NAD binding domain protein
DJ41_2052		1.7	0.00397	phenylacetic acid degradation B family protein
DJ41_2053	*paaA*	1.6	0.0059	phenylacetic acid degradation protein paaA
DJ41_368	*tynA*	1.4	0.02724	primary amine oxidase
Styrene degradation				
DJ41_358		7.4	4.9E-21	amidase family protein
DJ41_3063	*maiA*	1.3	0.0324	maleylacetoacetate isomerase
Tryptophan metabolism				
DJ41_358		7.4	4.9E-21	amidase family protein
DJ41_568	*lpdA*	4.6	2.7E-11	dihydrolipoyl dehydrogenase
DJ41_3252		3.6	1E-07	enoyl CoA hydratase isomerase family protein
DJ41_2048		2.7	1.8E-05	enoyl CoA hydratase isomerase family protein
DJ41_1959		2.0	0.00122	catalase family protein

DJ41_459	*mdlY*	1.4	0.03696	mandelamide hydrolase
DJ41_179		-2.1	0.00061	acetyl CoA C acetyltransferase family protein
Biosynthesis of cofactors				
DJ41_572	*lipA*	6.3	5.3E-17	lipoyl synthase
DJ41_364		5.1	5.7E-13	short chain dehydrogenase family protein
DJ41_568	*lpdA*	4.6	2.7E-11	dihydrolipoyl dehydrogenase
DJ41_2580		1.6	0.01903	homoserine dehydrogenase NAD binding domain protein
DJ41_180		-1.3	0.03778	short chain dehydrogenase family protein
Lipoic acid metabolism				
DJ41_572	*lipA*	6.3	5.3E-17	lipoyl synthase
Valine, leucine and isoleucine degradation				
DJ41_3255	*mmsB*	5.5	2.6E-14	3 hydroxyisobutyrate dehydrogenase
DJ41_3256	*mmsA*	5.4	2.7E-14	methylmalonate semialdehyde dehydrogenase
DJ41_568	*lpdA*	4.6	2.7E-11	dihydrolipoyl dehydrogenase
DJ41_3253		4.2	1.4E-09	hypothetical protein
DJ41_3252		3.6	1E-07	enoyl CoA hydratase isomerase family protein
DJ41_334	*pcaF*	3.2	1E-06	3 oxoadipyl CoA thiolase
DJ41_2045	*pcaF*	3.1	1.2E-06	3 oxoadipyl CoA thiolase
DJ41_2048		2.7	1.8E-05	enoyl CoA hydratase isomerase family protein
DJ41_179		-2.1	0.00061	acetyl CoA C acetyltransferase family protein
beta-Alanine metabolism				
DJ41_3256	*mmsA*	5.4	2.7E-14	methylmalonate semialdehyde dehydrogenase
DJ41_2745	*gabT*	5.0	6.6E-13	4 aminobutyrate transaminase
DJ41_3253		4.2	1.4E-09	hypothetical protein
DJ41_3252		3.6	1E-07	enoyl CoA hydratase isomerase family protein
DJ41_2048		2.7	1.8E-05	enoyl CoA hydratase isomerase family protein
DJ41_368	*tynA*	1.4	0.02724	primary amine oxidase
Carbon metabolism				
DJ41_3256	*mmsA*	5.4	2.7E-14	methylmalonate semialdehyde dehydrogenase
DJ41_3254		5.1	7E-13	AMP binding enzyme family protein
DJ41_569		4.8	3.4E-12	e3 binding domain protein
DJ41_568	*lpdA*	4.6	2.7E-11	dihydrolipoyl dehydrogenase
DJ41_3253		4.2	1.4E-09	hypothetical protein
DJ41_2046		2.9	5.1E-06	putative 3 hydroxybutyryl CoA dehydrogenase
DJ41_87		2.0	0.00097	AMP binding enzyme family protein
DJ41_442		2.0	0.00138	phosphoribosyl transferase domain protein

DJ41_1959		2.0	0.00122	catalase family protein
DJ41_1993		1.8	0.02469	3 hydroxyacyl CoA dehydrogenase NAD binding domain protein
DJ41_3165	*acs*	1.5	0.01024	acetate CoA ligase
DJ41_881	*prs*	1.3	0.02943	ribose phosphate diphosphokinase family protein
DJ41_2796		-1.2	0.04257	succinate CoA transferase family protein
DJ41_106	*acnB*	-1.4	0.01834	aconitate hydratase 2
DJ41_179		-2.1	0.00061	acetyl CoA C acetyltransferase family protein
DJ41_2528		-2.3	0.00025	isocitrate lyase
Inositol phosphate metabolism				
DJ41_3256	*mmsA*	5.4	2.7E-14	methylmalonate semialdehyde dehydrogenase
Propanoate metabolism				
DJ41_3256	*mmsA*	5.4	2.7E-14	methylmalonate semialdehyde dehydrogenase
DJ41_3254		5.1	7E-13	AMP binding enzyme family protein
DJ41_2745	*gabT*	5.0	6.6E-13	4 aminobutyrate transaminase
DJ41_568	*lpdA*	4.6	2.7E-11	dihydrolipoyl dehydrogenase
DJ41_3253		4.2	1.4E-09	hypothetical protein
DJ41_3252		3.6	1E-07	enoyl CoA hydratase isomerase family protein
DJ41_2048		2.7	1.8E-05	enoyl CoA hydratase isomerase family protein
DJ41_87		2.0	0.00097	AMP binding enzyme family protein
DJ41_3165	*acs*	1.5	0.01024	acetate CoA ligase
DJ41_106	*acnB*	-1.4	0.01834	aconitate hydratase 2
Biotin metabolism				
DJ41_364		5.1	5.7E-13	short chain dehydrogenase family protein
DJ41_180		-1.3	0.03778	short chain dehydrogenase family protein
Carbon fixation pathways in prokaryotes				
DJ41_3254		5.1	7E-13	AMP binding enzyme family protein
DJ41_87		2.0	0.00097	AMP binding enzyme family protein
DJ41_3165	*acs*	1.5	0.01024	acetate CoA ligase
DJ41_106	*acnB*	-1.4	0.01834	aconitate hydratase 2
DJ41_179		-2.1	0.00061	acetyl CoA C acetyltransferase family protein
Fatty acid biosynthesis				
DJ41_364		5.1	5.7E-13	short chain dehydrogenase family protein
DJ41_405		1.8	0.00765	AMP binding enzyme family protein
DJ41_180		-1.3	0.03778	short chain dehydrogenase family protein
Fatty acid metabolism				
DJ41_364		5.1	5.7E-13	short chain dehydrogenase family protein

DJ41_3253		4.2	1.4E-09	hypothetical protein
DJ41_3252		3.6	1E-07	enoyl CoA hydratase isomerase family protein
DJ41_334	*pcaF*	3.2	1E-06	3 oxoadipyl CoA thiolase
DJ41_2045	*pcaF*	3.1	1.2E-06	3 oxoadipyl CoA thiolase
DJ41_2048		2.7	1.8E-05	enoyl CoA hydratase isomerase family protein
DJ41_405		1.8	0.00765	AMP binding enzyme family protein
DJ41_180		-1.3	0.03778	short chain dehydrogenase family protein
DJ41_179		-2.1	0.00061	acetyl CoA C acetyltransferase family protein
Glycolysis / Gluconeogenesis				
DJ41_3254		5.1	7E-13	AMP binding enzyme family protein
DJ41_569		4.8	3.4E-12	e3 binding domain protein
DJ41_568	*lpdA*	4.6	2.7E-11	dihydrolipoyl dehydrogenase
DJ41_136		4.3	2.3E-10	iron containing alcohol dehydrogenase family protein
DJ41_133		3.0	2.5E-06	aldehyde dehydrogenase family protein
DJ41_87		2.0	0.00097	AMP binding enzyme family protein
DJ41_3165	*acs*	1.5	0.01024	acetate CoA ligase
Glyoxylate and dicarboxylate metabolism				
DJ41_3254		5.1	7E-13	AMP binding enzyme family protein
DJ41_568	*lpdA*	4.6	2.7E-11	dihydrolipoyl dehydrogenase
DJ41_87		2.0	0.00097	AMP binding enzyme family protein
DJ41_1959		2.0	0.00122	catalase family protein
DJ41_3165	*acs*	1.5	0.01024	acetate CoA ligase
DJ41_106	*acnB*	-1.4	0.01834	aconitate hydratase 2
DJ41_179		-2.1	0.00061	acetyl CoA C acetyltransferase family protein
DJ41_2528		-2.3	0.00025	isocitrate lyase
Methane metabolism				
DJ41_3254		5.1	7E-13	AMP binding enzyme family protein
DJ41_87		2.0	0.00097	AMP binding enzyme family protein
DJ41_3165	*acs*	1.5	0.01024	acetate CoA ligase
Prodigiosin biosynthesis				
DJ41_364		5.1	5.7E-13	short chain dehydrogenase family protein
DJ41_180		-1.3	0.03778	short chain dehydrogenase family protein
Pyruvate metabolism				
DJ41_3254		5.1	7E-13	AMP binding enzyme family protein
DJ41_569		4.8	3.4E-12	e3 binding domain protein
DJ41_568	*lpdA*	4.6	2.7E-11	dihydrolipoyl dehydrogenase

DJ41_136		4.3	2.3E-10	iron containing alcohol dehydrogenase family protein
DJ41_133		3.0	2.5E-06	aldehyde dehydrogenase family protein
DJ41_87		2.0	0.00097	AMP binding enzyme family protein
DJ41_3165	*acs*	1.5	0.01024	acetate CoA ligase
DJ41_2796		-1.2	0.04257	succinate CoA transferase family protein
DJ41_179		-2.1	0.00061	acetyl CoA C acetyltransferase family protein
Alanine, aspartate and glutamate metabolism				
DJ41_2745	*gabT*	5.0	6.6E-13	4 aminobutyrate transaminase
DJ41_544		2.8	7.2E-06	fumarase C family protein
DJ41_2746		2.1	0.00057	succinate semialdehyde dehydrogenase family protein
DJ41_2718	*gabD*	1.3	0.0364	succinate semialdehyde dehydrogenase
DJ41_803	*ansB*	-2.3	0.0002	glutaminase asparaginase
Butanoate metabolism				
DJ41_2745	*gabT*	5.0	6.6E-13	4 aminobutyrate transaminase
DJ41_3253		4.2	1.4E-09	hypothetical protein
DJ41_567	*budC*	4.2	8.2E-10	diacetyl reductase S acetoin forming
DJ41_3252		3.6	1E-07	enoyl CoA hydratase isomerase family protein
DJ41_566		3.5	1.2E-07	zinc binding dehydrogenase family protein
DJ41_2046		2.9	5.1E-06	putative 3 hydroxybutyryl CoA dehydrogenase
DJ41_2048		2.7	1.8E-05	enoyl CoA hydratase isomerase family protein
DJ41_2746		2.1	0.00057	succinate semialdehyde dehydrogenase family protein
DJ41_1993		1.8	0.02469	3 hydroxyacyl CoA dehydrogenase NAD binding domain protein
DJ41_2578		1.8	0.00739	thiamine pyrophosphate enzyme central domain protein
DJ41_2718	*gabD*	1.3	0.0364	succinate semialdehyde dehydrogenase
DJ41_2796		-1.2	0.04257	succinate CoA transferase family protein
DJ41_528		-1.3	0.03378	3 hydroxybutyrate dehydrogenase family protein
DJ41_179		-2.1	0.00061	acetyl CoA C acetyltransferase family protein
Citrate cycle (TCA cycle)				
DJ41_569		4.8	3.4E-12	e3 binding domain protein
DJ41_568	*lpdA*	4.6	2.7E-11	dihydrolipoyl dehydrogenase
DJ41_2796		-1.2	0.04257	succinate CoA transferase family protein
DJ41_106	*acnB*	-1.4	0.01834	aconitate hydratase 2

Glycine, serine and threonine metabolism				
DJ41_568	*lpdA*	4.6	2.7E-11	dihydrolipoyl dehydrogenase
DJ41_2409		3.2	6.2E-07	acyl CoA reductase family protein
DJ41_2579		1.8	0.00592	aldehyde dehydrogenase family protein
DJ41_368	*tynA*	1.4	0.02724	primary amine oxidase
Lysine degradation				
DJ41_568	*lpdA*	4.6	2.7E-11	dihydrolipoyl dehydrogenase
DJ41_3252		3.6	1E-07	enoyl CoA hydratase isomerase family protein
DJ41_2048		2.7	1.8E-05	enoyl CoA hydratase isomerase family protein
DJ41_2746		2.1	0.00057	succinate semialdehyde dehydrogenase family protein
DJ41_2718	*gabD*	1.3	0.0364	succinate semialdehyde dehydrogenase
DJ41_179		-2.1	0.00061	acetyl CoA C acetyltransferase family protein
Chloroalkane and chloroalkene degradation				
DJ41_136		4.3	2.3E-10	iron containing alcohol dehydrogenase family protein
Degradation of aromatic compounds				
DJ41_136		4.3	2.3E-10	iron containing alcohol dehydrogenase family protein
DJ41_338	*pcaC*	3.4	1.4E-06	4 carboxymuconolactone decarboxylase
DJ41_336	*pcaD*	3.2	1.9E-06	3 oxoadipate enol lactonase
DJ41_335	*pcaB*	3.0	3.6E-06	3 carboxy cis-cis muconate cycloisomerase
DJ41_340	*pcaG*	3.0	1.3E-05	protocatechuate 3 4 dioxygenase alpha subunit
DJ41_339	*pcaH*	2.9	1.7E-05	protocatechuate 3 4 dioxygenase beta subunit
DJ41_2585		1.9	0.00565	NAD P binding family protein
Fatty acid degradation				
DJ41_136		4.3	2.3E-10	iron containing alcohol dehydrogenase family protein
DJ41_3253		4.2	1.4E-09	hypothetical protein
DJ41_3252		3.6	1E-07	enoyl CoA hydratase isomerase family protein
DJ41_334	*pcaF*	3.2	1E-06	3 oxoadipyl CoA thiolase
DJ41_2045	*pcaF*	3.1	1.2E-06	3 oxoadipyl CoA thiolase
DJ41_2048		2.7	1.8E-05	enoyl CoA hydratase isomerase family protein
DJ41_2585		1.9	0.00565	NAD P binding family protein
DJ41_405		1.8	0.00765	AMP binding enzyme family protein
DJ41_179		-2.1	0.00061	acetyl CoA C acetyltransferase family protein
Naphthalene degradation				

DJ41_136		4.3	2.3E-10	iron containing alcohol dehydrogenase family protein
Tyrosine metabolism				
DJ41_136		4.3	2.3E-10	iron containing alcohol dehydrogenase family protein
DJ41_366		4.2	6.3E-10	flavin reductase like domain protein
DJ41_2746		2.1	0.00057	succinate semialdehyde dehydrogenase family protein
DJ41_368	*tynA*	1.4	0.02724	primary amine oxidase
DJ41_2718	*gabD*	1.3	0.0364	succinate semialdehyde dehydrogenase
DJ41_3063	*maiA*	1.3	0.0324	maleylacetoacetate isomerase
Riboflavin metabolism				
DJ41_366		4.2	6.3E-10	flavin reductase like domain protein
Starch and sucrose metabolism				
DJ41_906	*otsB*	3.8	2.1E-08	trehalose phosphatase
DJ41_907		3.2	4.5E-07	glycosyltransferase 20 family protein
Benzoate degradation				
DJ41_3252		3.6	1E-07	enoyl CoA hydratase isomerase family protein
DJ41_338	*pcaC*	3.4	1.4E-06	4 carboxymuconolactone decarboxylase
DJ41_332	*catI*	3.4	5.1E-07	3 oxoadipate CoA transferase subunit A
DJ41_333	*catJ*	3.2	1.2E-06	3 oxoadipate CoA transferase subunit B
DJ41_334	*pcaF*	3.2	1E-06	3 oxoadipyl CoA thiolase
DJ41_336	*pcaD*	3.2	1.9E-06	3 oxoadipate enol lactonase
DJ41_2045	*pcaF*	3.1	1.2E-06	3 oxoadipyl CoA thiolase
DJ41_335	*pcaB*	3.0	3.6E-06	3 carboxy cis-cis muconate cycloisomerase
DJ41_340	*pcaG*	3.0	1.3E-05	protocatechuate 3 4 dioxygenase alpha subunit
DJ41_339	*pcaH*	2.9	1.7E-05	protocatechuate 3 4 dioxygenase beta subunit
DJ41_2046		2.9	5.1E-06	putative 3 hydroxybutyryl CoA dehydrogenase
DJ41_2048		2.7	1.8E-05	enoyl CoA hydratase isomerase family protein
DJ41_1993		1.8	0.02469	3 hydroxyacyl CoA dehydrogenase NAD binding domain protein
DJ41_375	*catJ*	1.4	0.03146	3 oxoadipate CoA transferase subunit B
DJ41_179		-2.1	0.00061	acetyl CoA C acetyltransferase family protein
Caprolactam degradation				
DJ41_3252		3.6	1E-07	enoyl CoA hydratase isomerase family protein
DJ41_2048		2.7	1.8E-05	enoyl CoA hydratase isomerase family protein
DJ41_2001		2.0	0.00537	hypothetical protein

Geraniol degradation				
DJ41_3252		3.6	1E-07	enoyl CoA hydratase isomerase family protein
DJ41_334	*pcaF*	3.2	1E-06	3 oxoadipyl CoA thiolase
DJ41_2045	*pcaF*	3.1	1.2E-06	3 oxoadipyl CoA thiolase
DJ41_2048		2.7	1.8E-05	enoyl CoA hydratase isomerase family protein
DJ41_1247		-1.3	0.04616	AMP binding enzyme family protein
Limonene and pinene degradation				
DJ41_3252		3.6	1E-07	enoyl CoA hydratase isomerase family protein
DJ41_2048		2.7	1.8E-05	enoyl CoA hydratase isomerase family protein
Nicotinate and nicotinamide metabolism				
DJ41_1961		3.4	0.00011	competence damaged family protein
DJ41_2746		2.1	0.00057	succinate semialdehyde dehydrogenase family protein
DJ41_2580		1.6	0.01903	homoserine dehydrogenase NAD binding domain protein
DJ41_443		1.4	0.02103	nicotinate phosphoribosyltransferase family protein
DJ41_2718	*gabD*	1.3	0.0364	succinate semialdehyde dehydrogenase
alpha-Linolenic acid metabolism				
DJ41_334	*pcaF*	3.2	1E-06	3 oxoadipyl CoA thiolase
DJ41_2045	*pcaF*	3.1	1.2E-06	3 oxoadipyl CoA thiolase
Ethylbenzene degradation				
DJ41_334	*pcaF*	3.2	1E-06	3 oxoadipyl CoA thiolase
DJ41_2045	*pcaF*	3.1	1.2E-06	3 oxoadipyl CoA thiolase
Nucleotide metabolism				
DJ41_2077		3.2	2.9E-06	cytidine and deoxycytidylate deaminase zinc binding region family protein
DJ41_1568		1.5	0.0142	putative deoxyguanosinetriphosphate triphosphohydrolase
Penicillin and cephalosporin biosynthesis				
DJ41_372		3.2	0.00011	penicillin amidase family protein
DJ41_371		2.0	0.02353	penicillin amidase family protein
Purine metabolism				
DJ41_2077		3.2	2.9E-06	cytidine and deoxycytidylate deaminase zinc binding region family protein
DJ41_442		2.0	0.00138	phosphoribosyl transferase domain protein

DJ41_1568		1.5	0.0142	putative deoxyguanosinetriphosphate triphosphohydrolase
DJ41_881	*prs*	1.3	0.02943	ribose phosphate diphosphokinase family protein
DJ41_2522	*ureC*	-1.4	0.02276	urease alpha subunit
Biosynthesis of amino acids				
DJ41_341	*quiB*	3.1	1.4E-05	catabolic 3 dehydroquinate dehydratase
DJ41_442		2.0	0.00138	phosphoribosyl transferase domain protein
DJ41_2578		1.8	0.00739	thiamine pyrophosphate enzyme central domain protein
DJ41_2231		1.4	0.01821	pyrroline 5 carboxylate reductase dimerization family protein
DJ41_881	*prs*	1.3	0.02943	ribose phosphate diphosphokinase family protein
DJ41_106	*acnB*	-1.4	0.01834	aconitate hydratase 2
Phenylalanine, tyrosine and tryptophan biosynthesis				
DJ41_341	*quiB*	3.1	1.4E-05	catabolic 3 dehydroquinate dehydratase
DJ41_344	*quiA*	1.9	0.00262	quinate shikimate dehydrogenase
Polycyclic aromatic hydrocarbon degradation				
DJ41_340	*pcaG*	3.0	1.3E-05	protocatechuate 3 4 dioxygenase alpha subunit
DJ41_339	*pcaH*	2.9	1.7E-05	protocatechuate 3 4 dioxygenase beta subunit
Cysteine and methionine metabolism				
DJ41_3249		2.8	0.02373	autoinducer synthetase family protein
Quorum sensing				
DJ41_3249		2.8	0.02373	autoinducer synthetase family protein
DJ41_405		1.8	0.00765	AMP binding enzyme family protein
DJ41_1484		1.6	0.00995	hypothetical protein
DJ41_3582		1.6	0.00837	bacterial Ig like domain family protein
Biosynthesis of siderophore group nonribosomal peptides				
DJ41_342	*quiC*	2.6	6.1E-05	3 dehydroshikimate dehydratase
Arginine biosynthesis				
DJ41_803	*ansB*	-2.3	0.0002	glutaminase asparaginase
DJ41_2522	*ureC*	-1.4	0.02276	urease alpha subunit
D-Amino acid metabolism				
DJ41_803	*ansB*	-2.3	0.0002	glutaminase asparaginase
Fat digestion and absorption				
DJ41_179		-2.1	0.00061	acetyl CoA C acetyltransferase family protein
Terpenoid backbone biosynthesis				
DJ41_179		-2.1	0.00061	acetyl CoA C acetyltransferase family protein

Amyotrophic lateral sclerosis				
DJ41_1959		2.0	0.00122	catalase family protein
Chemical carcinogenesis - reactive oxygen species				
DJ41_1959		2.0	0.00122	catalase family protein
FoxO signalling pathway				
DJ41_1959		2.0	0.00122	catalase family protein
Glycerolipid metabolism				
DJ41_2867	*lip*	2.0	0.0018	lactonizing lipase
Longevity regulating pathway				
DJ41_1959		2.0	0.00122	catalase family protein
Longevity regulating pathway - multiple species				
DJ41_1959		2.0	0.00122	catalase family protein
Longevity regulating pathway - worm				
DJ41_1959		2.0	0.00122	catalase family protein
MAPK signaling pathway - plant				
DJ41_1959		2.0	0.00122	catalase family protein
MAPK signaling pathway - yeast				
DJ41_1959		2.0	0.00122	catalase family protein
Pathways of neurodegeneration - multiple diseases				
DJ41_1959		2.0	0.00122	catalase family protein
Pentose phosphate pathway				
DJ41_442		2.0	0.00138	phosphoribosyl transferase domain protein
DJ41_881	*prs*	1.3	0.02943	ribose phosphate diphosphokinase family protein
Peroxisome				
DJ41_1959		2.0	0.00122	catalase family protein
DJ41_405		1.8	0.00765	AMP binding enzyme family protein

Table S5. Differentially regulated genes in the Δ*gacSA* mutant strain versus the wild-type strain ATCC 19606 in gene ontology.

Category and locus tag	Gene	Log_2 fold change	p-value	Predicted function
Biological Process (BP)				
transmembrane transport				
DJ41_356		6.6	2.4E-06	organic Anion Transporter Polypeptide family protein
DJ41_1671		5.9	6.5E-06	amino acid permease family protein
DJ41_546		4.5	0.00035	sugar and other transporter family protein
DJ41_2743		4.4	0.00045	amino acid permease family protein
DJ41_132	*eat*	4.2	0.00094	ethanolamine permease
DJ41_337		4.1	0.00103	MFS transporter aromatic acid H symporter family protein
DJ41_2257		3.9	0.01145	MFS transporter aromatic acid H symporter family protein
DJ41_3250		3.7	0.00301	sugar and other transporter family protein
DJ41_3065	*aroP*	3.7	0.00231	aromatic amino acid transport protein AroP
DJ41_3174		2.9	0.01324	transporter solute sodium symporter family protein
DJ41_2413		2.9	0.0185	amino acid permease family protein
DJ41_776		2.9	0.0163	amino ABC transporter permease 3 TM region His Glu Gln Arg opine family domain protein
DJ41_3534		2.7	0.03541	membrane transport family protein
DJ41_1746		2.7	0.02599	amino acid peptide transporter family protein
DJ41_2576		2.6	0.03089	sugar and other transporter family protein
DJ41_184		2.3	0.04921	major Facilitator Superfamily protein
DJ41_3034	*ydhP*	-2.2	0.03085	inner membrane transport protein ydhP
cell redox homeostasis				
DJ41_568	*lpdA*	4.7	0.0002	dihydrolipoyl dehydrogenase
regulation of transcription, DNA-templated				
DJ41_520		3.3	0.02302	helix turn helix domain protein
DJ41_2411		2.9	0.01753	bacterial regulatory s *gntR* family protein
DJ41_367		2.8	0.02426	helix turn helix domain protein
amino acid transport				

DJ41_776		2.9	0.0163	amino ABC transporter permease 3 TM region His Glu Gln Arg opine family domain protein
tricarboxylic acid cycle				
DJ41_544		2.7	0.02441	fumarase C family protein
DNA replication				
DJ41_1811		-2.5	0.01752	hypothetical protein
protein folding				
DJ41_2868		2.4	0.04939	proteobacterial lipase chaperone family protein
Cellular Component (CC)				
membrane				
DJ41_1169	*tatB*	15.1	0.00198	twin arginine targeting protein translocase TatB
DJ41_2743		4.4	0.00045	amino acid permease family protein
DJ41_3065	*aroP*	3.7	0.00231	aromatic amino acid transport protein AroP
DJ41_3174		2.9	0.01324	transporter solute sodium symporter family protein
DJ41_2413		2.9	0.0185	amino acid permease family protein
DJ41_776		2.9	0.0163	amino ABC transporter permease 3 TM region His Glu Gln Arg opine family domain protein
DJ41_559		2.7	0.02625	*eamA* like transporter family protein
DJ41_1746		2.7	0.02599	amino acid peptide transporter family protein
DJ41_2123		2.5	0.03579	3 hydroxyacyl CoA dehydrogenase NAD binding domain protein
DJ41_406		2.5	0.04997	sodium Bile acid symporter family protein
DJ41_2868		2.4	0.04939	proteobacterial lipase chaperone family protein
DJ41_2903		-4.2	0.00027	*eamA* like transporter family protein
integral component of membrane				
DJ41_356		6.6	2.4E-06	organic Anion Transporter Polypeptide family protein
DJ41_546		4.5	0.00035	sugar and other transporter family protein
DJ41_337		4.1	0.00103	MFS transporter aromatic acid H symporter family protein
DJ41_95	*kdpB*	3.9	0.00141	K transporting ATPase B subunit
DJ41_2257		3.9	0.01145	MFS transporter aromatic acid H symporter family protein
DJ41_3250		3.7	0.00301	sugar and other transporter family protein
DJ41_343	*oprB*	3.4	0.00601	porin B
DJ41_96	*kdpC*	3.3	0.00644	K transporting ATPase C subunit

DJ41_776		2.9	0.0163	amino ABC transporter permease 3 TM region His Glu Gln Arg opine family domain protein
DJ41_559		2.7	0.02625	*eamA* like transporter family protein
DJ41_3534		2.7	0.03541	membrane transport family protein
DJ41_2576		2.6	0.03089	sugar and other transporter family protein
DJ41_3022		2.6	0.03348	sulfite exporter TauE SafE family protein
DJ41_2256		2.6	0.04816	benzoate transporter family protein
DJ41_184		2.3	0.04921	major Facilitator Superfamily protein
DJ41_3034	*ydhP*	-2.2	0.03085	inner membrane transport protein ydhP
DJ41_2903		-4.2	0.00027	eamA like transporter family protein
outer membrane				
DJ41_1773		3.1	0.01119	*ompW* family protein
Molecular Function (MF)				
flavin adenine dinucleotide binding				
DJ41_359		9.2	1.4E-09	hypothetical protein
DJ41_3253		3.4	0.00472	hypothetical protein
DJ41_2001		3.2	0.01164	hypothetical protein
oxidoreductase activity, acting on the CH-CH group of donors				
DJ41_359		9.2	1.4E-09	hypothetical protein
DJ41_3253		3.4	0.00472	hypothetical protein
DJ41_2001		3.2	0.01164	hypothetical protein
4 iron, 4 sulfur cluster binding				
DJ41_572	*lipA*	6.4	2.2E-06	lipoyl synthase
catalytic activity				
DJ41_572	*lipA*	6.4	2.2E-06	lipoyl synthase
DJ41_1670		5.7	1.3E-05	thiamine pyrophosphate enzyme central domain protein
DJ41_2048		5.3	3.9E-05	enoyl CoA hydratase isomerase family protein
DJ41_2044	*paaF*	5.0	7E-05	phenylacetate CoA ligase
DJ41_3254		5.0	8.3E-05	AMP binding enzyme family protein
DJ41_2047	*paaB*	5.0	8E-05	phenylacetate degradation putative enoyl CoA hydratase PaaB
DJ41_906	*otsB*	3.4	0.0054	trehalose phosphatase
DJ41_3252		3.1	0.00976	enoyl CoA hydratase isomerase family protein
DJ41_340	*pcaG*	3.0	0.01302	protocatechuate 3 4 dioxygenase alpha subunit
DJ41_339	*pcaH*	3.0	0.01529	protocatechuate 3 4 dioxygenase beta subunit
DJ41_3064	*fahA*	2.8	0.01737	fumarylacetoacetase

DJ41_405		2.7	0.03062	AMP binding enzyme family protein
DJ41_1070		2.6	0.02715	AMP binding enzyme family protein
DJ41_377	*catA*	2.4	0.0446	catechol 1 2 dioxygenase
iron-sulfur cluster binding				
DJ41_572	*lipA*	6.4	2.2E-06	lipoyl synthase
DJ41_365		5.0	9.3E-05	2Fe 2S iron sulfur cluster binding domain protein
DJ41_2049		4.8	0.00015	2Fe 2S iron sulfur cluster binding domain protein
DJ41_2254	*benC*	4.6	0.0003	benzoate 1 2 dioxygenase electron transfer component
oxidoreductase activity				
DJ41_3256	*mmsA*	6.4	1.8E-06	methylmalonate semialdehyde dehydrogenase
DJ41_136		5.9	8.1E-06	iron containing alcohol dehydrogenase family protein
DJ41_362		5.7	1.3E-05	ring hydroxylating alpha subunit family protein
DJ41_2046		5.4	2.8E-05	putative 3 hydroxybutyryl CoA dehydrogenase
DJ41_2252	*benA*	5.1	7.6E-05	benzoate 1 2 dioxygenase large subunit
DJ41_365		5.0	9.3E-05	2Fe 2S iron sulfur cluster binding domain protein
DJ41_2049		4.8	0.00015	2Fe 2S iron sulfur cluster binding domain protein
DJ41_568	*lpdA*	4.7	0.0002	dihydrolipoyl dehydrogenase
DJ41_2254	*benC*	4.6	0.0003	benzoate 1 2 dioxygenase electron transfer component
DJ41_2054	*paaN*	3.8	0.00186	phenylacetic acid degradation protein paaN
DJ41_133		3.7	0.00233	aldehyde dehydrogenase family protein
DJ41_1668		3.5	0.00395	aldehyde dehydrogenase family protein
DJ41_2409		3.3	0.00652	acyl CoA reductase family protein
DJ41_2590		2.9	0.03081	rieske 2Fe 2S domain protein
DJ41_2131		2.5	0.03579	3 hydroxyacyl CoA dehydrogenase NAD binding domain protein
DJ41_3035		-2.7	0.01125	aldo keto reductase family protein
metal ion binding				
DJ41_136		5.9	8.1E-06	iron containing alcohol dehydrogenase family protein
2 iron, 2 sulfur cluster binding				
DJ41_362		5.7	1.3E-05	ring hydroxylating alpha subunit family protein
DJ41_2252	*benA*	5.1	7.6E-05	benzoate 1 2 dioxygenase large subunit
DJ41_2590		2.9	0.03081	rieske 2Fe 2S domain protein
iron ion binding				

DJ41_362		5.7	1.3E-05	ring hydroxylating alpha subunit family protein
DJ41_2252	*benA*	5.1	7.6E-05	benzoate 1 2 dioxygenase large subunit
DJ41_377	*catA*	2.4	0.0446	catechol 1 2 dioxygenase
magnesium ion binding				
DJ41_1670		5.7	1.3E-05	thiamine pyrophosphate enzyme central domain protein
DJ41_881	*prs*	2.3	0.04751	ribose phosphate diphosphokinase family protein
NAD binding				
DJ41_3255	*mmsB*	5.7	1.5E-05	3 hydroxyisobutyrate dehydrogenase
NADP binding				
DJ41_3255	*mmsB*	5.7	1.5E-05	3 hydroxyisobutyrate dehydrogenase
acyltransferase activity, transferring groups other than amino-acyl groups				
DJ41_2045	*pcaF*	5.2	4.8E-05	3 oxoadipyl CoA thiolase
DJ41_334	*pcaF*	4.0	0.00129	3 oxoadipyl CoA thiolase
CoA-transferase activity				
DJ41_332	*catI*	5.0	0.00011	3 oxoadipate CoA transferase subunit A
DJ41_333	*catJ*	4.4	0.00043	3 oxoadipate CoA transferase subunit B
DJ41_376	*catI*	2.9	0.01927	3 oxoadipate CoA transferase subunit A
DJ41_375	*catJ*	2.5	0.04201	3 oxoadipate CoA transferase subunit B
electron transfer activity				
DJ41_365		5.0	9.3E-05	2Fe 2S iron sulfur cluster binding domain protein
DJ41_2049		4.8	0.00015	2Fe 2S iron sulfur cluster binding domain protein
DJ41_2254	*benC*	4.6	0.0003	benzoate 1 2 dioxygenase electron transfer component
acyltransferase activity				
DJ41_569		4.6	0.00023	e3 binding domain protein
transmembrane transporter activity				
DJ41_546		4.5	0.00035	sugar and other transporter family protein
DJ41_132	*eat*	4.2	0.00094	ethanolamine permease
DJ41_3174		2.9	0.01324	transporter solute sodium symporter family protein
DJ41_1746		2.7	0.02599	amino acid peptide transporter family protein
FMN binding				
DJ41_366		4.2	0.00076	flavin reductase like domain protein
pyridoxal phosphate binding				
DJ41_2745	*gabT*	4.2	0.0007	4 aminobutyrate transaminase
ATP binding				
DJ41_95	*kdpB*	3.9	0.00141	K transporting ATPase B subunit

DJ41_1811		-2.5	0.01752	hypothetical protein
protein binding				
DJ41_3063	*maiA*	3.8	0.00177	maleylacetoacetate isomerase
DNA-binding transcription factor activity				
DJ41_520		3.3	0.02302	helix turn helix domain protein
DJ41_2411		2.9	0.01753	bacterial regulatory s *gntR* family protein
DJ41_367		2.8	0.02426	helix turn helix domain protein
hydrolase activity				
DJ41_372		3.3	0.01435	penicillin amidase family protein
sequence-specific DNA binding				
DJ41_520		3.3	0.02302	helix turn helix domain protein
DJ41_367		2.8	0.02426	helix turn helix domain protein
lyase activity				
DJ41_544		2.7	0.02441	fumarase C family protein
DNA binding				
DJ41_769		2.6	0.03538	bacterial regulatory s tetR family protein
DJ41_197		2.4	0.04946	bacterial regulatory s tetR family protein
DNA helicase activity				
DJ41_1811		-2.5	0.01752	hypothetical protein
nucleic acid binding				
DJ41_2233		2.4	0.04352	Cold shock DNA binding domain protein
unfolded protein binding				
DJ41_2868		2.4	0.04939	proteobacterial lipase chaperone family protein
methyltransferase activity				
DJ41_2339		-2.3	0.03236	C 5 cytosine specific DNA methylase family protein

Table S6. Differentially regulated genes in the Δ*gacSA* mutant strain versus the wild-type strain ATCC 19606 in KEGG.

Category and locus tag	Gene	Log_2 fold change	p-value	Predicted function
Bacterial secretion system				
DJ41_1169	*tatB*	15.1	0.00198	twin arginine targeting protein translocase TatB
Protein export				
DJ41_1169	*tatB*	15.1	0.00198	twin arginine targeting protein translocase TatB
Aminobenzoate degradation				
DJ41_358		6.9	4.5E-07	amidase family protein
DJ41_2048		5.3	3.9E-05	enoyl CoA hydratase isomerase family protein
DJ41_3252		3.1	0.00976	enoyl CoA hydratase isomerase family protein
Arginine and proline metabolism				
DJ41_358		6.9	4.5E-07	amidase family protein
DJ41_2231		2.5	0.03243	pyrroline 5 carboxylate reductase dimerization family protein
Phenylalanine metabolism				
DJ41_358		6.9	4.5E-07	amidase family protein
DJ41_2041		5.6	1.9E-05	phenylacetic acid degradation protein *paaI*
DJ41_2046		5.4	2.8E-05	putative 3 hydroxybutyryl CoA dehydrogenase
DJ41_2048		5.3	3.9E-05	enoyl CoA hydratase isomerase family protein
DJ41_2044	*paaF*	5.0	7E-05	phenylacetate CoA ligase
DJ41_2047	*paaB*	5.0	8E-05	phenylacetate degradation putative enoyl CoA hydratase PaaB
DJ41_2049		4.8	0.00015	2Fe 2S iron sulfur cluster binding domain protein
DJ41_2050	*paaJ*	4.7	0.00017	phenylacetate CoA oxygenase PaaJ subunit
DJ41_2051	*paaC*	4.5	0.00027	phenylacetic acid degradation protein paaC
DJ41_2053	*paaA*	4.3	0.00051	phenylacetic acid degradation protein paaA
DJ41_2052		4.3	0.00053	phenylacetic acid degradation B family protein
DJ41_2054	*paaN*	3.8	0.00186	phenylacetic acid degradation protein paaN
DJ41_3252		3.1	0.00976	enoyl CoA hydratase isomerase family protein
DJ41_3060	*hppD*	2.3	0.04994	4 hydroxyphenylpyruvate dioxygenase
Styrene degradation				
DJ41_358		6.9	4.5E-07	amidase family protein
DJ41_3063	*maiA*	3.8	0.00177	maleylacetoacetate isomerase

DJ41_3064	*fahA*	2.8	0.01737	fumarylacetoacetase
Tryptophan metabolism				
DJ41_358		6.9	4.5E-07	amidase family protein
DJ41_2048		5.3	3.9E-05	enoyl CoA hydratase isomerase family protein
DJ41_568	*lpdA*	4.7	0.0002	dihydrolipoyl dehydrogenase
DJ41_3252		3.1	0.00976	enoyl CoA hydratase isomerase family protein
beta-Alanine metabolism				
DJ41_3256	*mmsA*	6.4	1.8E-06	methylmalonate semialdehyde dehydrogenase
DJ41_2048		5.3	3.9E-05	enoyl CoA hydratase isomerase family protein
DJ41_2745	*gabT*	4.2	0.0007	4 aminobutyrate transaminase
DJ41_3253		3.4	0.00472	hypothetical protein
DJ41_3252		3.1	0.00976	enoyl CoA hydratase isomerase family protein
Biosynthesis of cofactors				
DJ41_572	*lipA*	6.4	2.2E-06	lipoyl synthase
DJ41_568	*lpdA*	4.7	0.0002	dihydrolipoyl dehydrogenase
DJ41_364		4.6	0.00028	short chain dehydrogenase family protein
DJ41_3060	*hppD*	2.3	0.04994	4 hydroxyphenylpyruvate dioxygenase
Carbon metabolism				
DJ41_3256	*mmsA*	6.4	1.8E-06	methylmalonate semialdehyde dehydrogenase
DJ41_2046		5.4	2.8E-05	putative 3 hydroxybutyryl CoA dehydrogenase
DJ41_3254		5.0	8.3E-05	AMP binding enzyme family protein
DJ41_568	*lpdA*	4.7	0.0002	dihydrolipoyl dehydrogenase
DJ41_569		4.6	0.00023	e3 binding domain protein
DJ41_3253		3.4	0.00472	hypothetical protein
DJ41_881	*prs*	2.3	0.04751	ribose phosphate diphosphokinase family protein
Inositol phosphate metabolism				
DJ41_3256	*mmsA*	6.4	1.8E-06	methylmalonate semialdehyde dehydrogenase
Lipoic acid metabolism				
DJ41_572	*lipA*	6.4	2.2E-06	lipoyl synthase
Propanoate metabolism				
DJ41_3256	*mmsA*	6.4	1.8E-06	methylmalonate semialdehyde dehydrogenase
DJ41_2048		5.3	3.9E-05	enoyl CoA hydratase isomerase family protein
DJ41_3254		5.0	8.3E-05	AMP binding enzyme family protein
DJ41_568	*lpdA*	4.7	0.0002	dihydrolipoyl dehydrogenase
DJ41_2745	*gabT*	4.2	0.0007	4 aminobutyrate transaminase
DJ41_3253		3.4	0.00472	hypothetical protein
DJ41_3252		3.1	0.00976	enoyl CoA hydratase isomerase family protein

Valine, leucine and isoleucine degradation				
DJ41_3256	*mmsA*	6.4	1.8E-06	methylmalonate semialdehyde dehydrogenase
DJ41_3255	*mmsB*	5.7	1.5E-05	3 hydroxyisobutyrate dehydrogenase
DJ41_2048		5.3	3.9E-05	enoyl CoA hydratase isomerase family protein
DJ41_2045	*pcaF*	5.2	4.8E-05	3 oxoadipyl CoA thiolase
DJ41_568	*lpdA*	4.7	0.0002	dihydrolipoyl dehydrogenase
DJ41_334	*pcaF*	4.0	0.00129	3 oxoadipyl CoA thiolase
DJ41_3253		3.4	0.00472	hypothetical protein
DJ41_3252		3.1	0.00976	enoyl CoA hydratase isomerase family protein
Chloroalkane and chloroalkene degradation				
DJ41_136		5.9	8.1E-06	iron containing alcohol dehydrogenase family protein
Degradation of aromatic compounds				
DJ41_136		5.9	8.1E-06	iron containing alcohol dehydrogenase family protein
DJ41_2252	*benA*	5.1	7.6E-05	benzoate 1 2 dioxygenase large subunit
DJ41_2253	*benB*	4.7	0.00024	benzoate 1 2 dioxygenase small subunit
DJ41_2254	*benC*	4.6	0.0003	benzoate 1 2 dioxygenase electron transfer component
DJ41_336	*pcaD*	4.0	0.00132	3 oxoadipate enol lactonase
DJ41_335	*pcaB*	3.7	0.00225	3 carboxy cis-cis muconate cycloisomerase
DJ41_2010	*pobA*	3.7	0.00263	4 hydroxybenzoate 3 monooxygenase
DJ41_338	*pcaC*	3.2	0.00864	4 carboxymuconolactone decarboxylase
DJ41_340	*pcaG*	3.0	0.01302	protocatechuate 3 4 dioxygenase alpha subunit
DJ41_2255		3.0	0.0152	short chain dehydrogenase family protein
DJ41_339	*pcaH*	3.0	0.01529	protocatechuate 3 4 dioxygenase beta subunit
DJ41_379		2.8	0.0186	muconate and chloromuconate cycloisomerases family protein
DJ41_378	*catC*	2.7	0.03306	muconolactone delta isomerase
DJ41_377	*catA*	2.4	0.0446	catechol 1 2 dioxygenase
Fatty acid degradation				
DJ41_136		5.9	8.1E-06	iron containing alcohol dehydrogenase family protein
DJ41_2048		5.3	3.9E-05	enoyl CoA hydratase isomerase family protein
DJ41_2045	*pcaF*	5.2	4.8E-05	3 oxoadipyl CoA thiolase
DJ41_334	*pcaF*	4.0	0.00129	3 oxoadipyl CoA thiolase
DJ41_3253		3.4	0.00472	hypothetical protein

DJ41_3252		3.1	0.00976	enoyl CoA hydratase isomerase family protein
DJ41_405		2.7	0.03062	AMP binding enzyme family protein
Glycolysis / Gluconeogenesis				
DJ41_136		5.9	8.1E-06	iron containing alcohol dehydrogenase family protein
DJ41_3254		5.0	8.3E-05	AMP binding enzyme family protein
DJ41_568	*lpdA*	4.7	0.0002	dihydrolipoyl dehydrogenase
DJ41_569		4.6	0.00023	e3 binding domain protein
DJ41_133		3.7	0.00233	aldehyde dehydrogenase family protein
Naphthalene degradation				
DJ41_136		5.9	8.1E-06	iron containing alcohol dehydrogenase family protein
Pyruvate metabolism				
DJ41_136		5.9	8.1E-06	iron containing alcohol dehydrogenase family protein
DJ41_3254		5.0	8.3E-05	AMP binding enzyme family protein
DJ41_568	*lpdA*	4.7	0.0002	dihydrolipoyl dehydrogenase
DJ41_569		4.6	0.00023	e3 binding domain protein
DJ41_133		3.7	0.00233	aldehyde dehydrogenase family protein
Tyrosine metabolism				
DJ41_136		5.9	8.1E-06	iron containing alcohol dehydrogenase family protein
DJ41_366		4.2	0.00076	flavin reductase like domain protein
DJ41_3063	*maiA*	3.8	0.00177	maleylacetoacetate isomerase
DJ41_3064	*fahA*	2.8	0.01737	fumarylacetoacetase
DJ41_3060	*hppD*	2.3	0.04994	4 hydroxyphenylpyruvate dioxygenase
Benzoate degradation				
DJ41_2046		5.4	2.8E-05	putative 3 hydroxybutyryl CoA dehydrogenase
DJ41_2048		5.3	3.9E-05	enoyl CoA hydratase isomerase family protein
DJ41_2045	*pcaF*	5.2	4.8E-05	3 oxoadipyl CoA thiolase
DJ41_2252	*benA*	5.1	7.6E-05	benzoate 1 2 dioxygenase large subunit
DJ41_332	*catI*	5.0	0.00011	3 oxoadipate CoA transferase subunit A
DJ41_2253	*benB*	4.7	0.00024	benzoate 1 2 dioxygenase small subunit
DJ41_2254	*benC*	4.6	0.0003	benzoate 1 2 dioxygenase electron transfer component
DJ41_333	*catJ*	4.4	0.00043	3 oxoadipate CoA transferase subunit B
DJ41_336	*pcaD*	4.0	0.00132	3 oxoadipate enol lactonase

DJ41_334	*pcaF*	4.0	0.00129	3 oxoadipyl CoA thiolase
DJ41_335	*pcaB*	3.7	0.00225	3 carboxy cis-cis muconate cycloisomerase
DJ41_2010	*pobA*	3.7	0.00263	4 hydroxybenzoate 3 monooxygenase
DJ41_338	*pcaC*	3.2	0.00864	4 carboxymuconolactone decarboxylase
DJ41_3252		3.1	0.00976	enoyl CoA hydratase isomerase family protein
DJ41_340	*pcaG*	3.0	0.01302	protocatechuate 3 4 dioxygenase alpha subunit
DJ41_2255		3.0	0.0152	short chain dehydrogenase family protein
DJ41_339	*pcaH*	3.0	0.01529	protocatechuate 3 4 dioxygenase beta subunit
DJ41_376	*catI*	2.9	0.01927	3 oxoadipate CoA transferase subunit A
DJ41_379		2.8	0.0186	muconate and chloromuconate cycloisomerases family protein
DJ41_378	*catC*	2.7	0.03306	muconolactone delta isomerase
DJ41_375	*catJ*	2.5	0.04201	3 oxoadipate CoA transferase subunit B
DJ41_377	*catA*	2.4	0.0446	catechol 1 2 dioxygenase
Butanoate metabolism				
DJ41_2046		5.4	2.8E-05	putative 3 hydroxybutyryl CoA dehydrogenase
DJ41_2048		5.3	3.9E-05	enoyl CoA hydratase isomerase family protein
DJ41_2745	*gabT*	4.2	0.0007	4 aminobutyrate transaminase
DJ41_567	*budC*	3.9	0.00143	diacetyl reductase S acetoin forming
DJ41_3253		3.4	0.00472	hypothetical protein
DJ41_566		3.3	0.00678	zinc binding dehydrogenase family protein
DJ41_3252		3.1	0.00976	enoyl CoA hydratase isomerase family protein
Caprolactam degradation				
DJ41_2048		5.3	3.9E-05	enoyl CoA hydratase isomerase family protein
DJ41_2001		3.2	0.01164	hypothetical protein
DJ41_3252		3.1	0.00976	enoyl CoA hydratase isomerase family protein
Fatty acid metabolism				
DJ41_2048		5.3	3.9E-05	enoyl CoA hydratase isomerase family protein
DJ41_2045	*pcaF*	5.2	4.8E-05	3 oxoadipyl CoA thiolase
DJ41_364		4.6	0.00028	short chain dehydrogenase family protein
DJ41_334	*pcaF*	4.0	0.00129	3 oxoadipyl CoA thiolase
DJ41_3253		3.4	0.00472	hypothetical protein
DJ41_3252		3.1	0.00976	enoyl CoA hydratase isomerase family protein
DJ41_405		2.7	0.03062	AMP binding enzyme family protein
Geraniol degradation				
DJ41_2048		5.3	3.9E-05	enoyl CoA hydratase isomerase family protein
DJ41_2045	*pcaF*	5.2	4.8E-05	3 oxoadipyl CoA thiolase

DJ41_334	*pcaF*	4.0	0.00129	3 oxoadipyl CoA thiolase
DJ41_3252		3.1	0.00976	enoyl CoA hydratase isomerase family protein
Limonene and pinene degradation				
DJ41_2048		5.3	3.9E-05	enoyl CoA hydratase isomerase family protein
DJ41_3252		3.1	0.00976	enoyl CoA hydratase isomerase family protein
Lysine degradation				
DJ41_2048		5.3	3.9E-05	enoyl CoA hydratase isomerase family protein
DJ41_568	*lpdA*	4.7	0.0002	dihydrolipoyl dehydrogenase
DJ41_3252		3.1	0.00976	enoyl CoA hydratase isomerase family protein
alpha-Linolenic acid metabolism				
DJ41_2045	*pcaF*	5.2	4.8E-05	3 oxoadipyl CoA thiolase
DJ41_334	*pcaF*	4.0	0.00129	3 oxoadipyl CoA thiolase
Ethylbenzene degradation				
DJ41_2045	*pcaF*	5.2	4.8E-05	3 oxoadipyl CoA thiolase
DJ41_334	*pcaF*	4.0	0.00129	3 oxoadipyl CoA thiolase
Fluorobenzoate degradation				
DJ41_2252	*benA*	5.1	7.6E-05	benzoate 1 2 dioxygenase large subunit
DJ41_2253	*benB*	4.7	0.00024	benzoate 1 2 dioxygenase small subunit
DJ41_2254	*benC*	4.6	0.0003	benzoate 1 2 dioxygenase electron transfer component
DJ41_2255		3.0	0.0152	short chain dehydrogenase family protein
DJ41_379		2.8	0.0186	muconate and chloromuconate cycloisomerases family protein
DJ41_377	*catA*	2.4	0.0446	catechol 1 2 dioxygenase
Xylene degradation				
DJ41_2252	*benA*	5.1	7.6E-05	benzoate 1 2 dioxygenase large subunit
DJ41_2253	*benB*	4.7	0.00024	benzoate 1 2 dioxygenase small subunit
DJ41_2254	*benC*	4.6	0.0003	benzoate 1 2 dioxygenase electron transfer component
DJ41_2255		3.0	0.0152	short chain dehydrogenase family protein
Biofilm formation - Vibrio cholerae				
DJ41_2044	*paaF*	5.0	7E-05	phenylacetate CoA ligase
Carbon fixation pathways in prokaryotes				
DJ41_3254		5.0	8.3E-05	AMP binding enzyme family protein
Glyoxylate and dicarboxylate metabolism				
DJ41_3254		5.0	8.3E-05	AMP binding enzyme family protein
DJ41_568	*lpdA*	4.7	0.0002	dihydrolipoyl dehydrogenase

Methane metabolism				
DJ41_3254		5.0	8.3E-05	AMP binding enzyme family protein
Citrate cycle (TCA cycle)				
DJ41_568	*lpdA*	4.7	0.0002	dihydrolipoyl dehydrogenase
DJ41_569		4.6	0.00023	e3 binding domain protein
Glycine, serine and threonine metabolism				
DJ41_568	*lpdA*	4.7	0.0002	dihydrolipoyl dehydrogenase
DJ41_2409		3.3	0.00652	acyl CoA reductase family protein
Biotin metabolism				
DJ41_364		4.6	0.00028	short chain dehydrogenase family protein
Fatty acid biosynthesis				
DJ41_364		4.6	0.00028	short chain dehydrogenase family protein
DJ41_405		2.7	0.03062	AMP binding enzyme family protein
Prodigiosin biosynthesis				
DJ41_364		4.6	0.00028	short chain dehydrogenase family protein
Two-component system				
DJ41_94	*kdpA*	4.4	0.0005	K transporting ATPase A subunit
DJ41_95	*kdpB*	3.9	0.00141	K transporting ATPase B subunit
DJ41_96	*kdpC*	3.3	0.00644	K transporting ATPase C subunit
DJ41_776		2.9	0.0163	amino ABC transporter permease 3 TM region His Glu Gln Arg opine family domain protein
DJ41_777		2.6	0.0273	bacterial extracellular solute binding s 3 family protein
Alanine, aspartate and glutamate metabolism				
DJ41_2745	*gabT*	4.2	0.0007	4 aminobutyrate transaminase
DJ41_544		2.7	0.02441	fumarase C family protein
Riboflavin metabolism				
DJ41_366		4.2	0.00076	flavin reductase like domain protein
Starch and sucrose metabolism				
DJ41_906	*otsB*	3.4	0.0054	trehalose phosphatase
Penicillin and cephalosporin biosynthesis				
DJ41_372		3.3	0.01435	penicillin amidase family protein
Biosynthesis of amino acids				
DJ41_341	*quiB*	3.2	0.01099	catabolic 3 dehydroquinate dehydratase
DJ41_2231		2.5	0.03243	pyrroline 5 carboxylate reductase dimerization family protein
DJ41_881	*prs*	2.3	0.04751	ribose phosphate diphosphokinase family protein

Nicotinate and nicotinamide metabolism				
DJ41_1961		3.2	0.02072	competence damaged family protein
Phenylalanine, tyrosine and tryptophan biosynthesis				
DJ41_341	*quiB*	3.2	0.01099	catabolic 3 dehydroquinate dehydratase
Polycyclic aromatic hydrocarbon degradation				
DJ41_340	*pcaG*	3.0	0.01302	protocatechuate 3 4 dioxygenase alpha subunit
DJ41_339	*pcaH*	3.0	0.01529	protocatechuate 3 4 dioxygenase beta subunit
Chlorocyclohexane and chlorobenzene degradation				
DJ41_379		2.8	0.0186	muconate and chloromuconate cycloisomerases family protein
DJ41_377	*catA*	2.4	0.0446	catechol 1 2 dioxygenase
Toluene degradation				
DJ41_379		2.8	0.0186	muconate and chloromuconate cycloisomerases family protein
DJ41_377	*catA*	2.4	0.0446	catechol 1 2 dioxygenase
Adipocytokine signaling pathway				
DJ41_405		2.7	0.03062	AMP binding enzyme family protein
Biosynthesis of siderophore group nonribosomal peptides				
DJ41_342	*quiC*	2.7	0.02402	3 dehydroshikimate dehydratase
Ferroptosis				
DJ41_405		2.7	0.03062	AMP binding enzyme family protein
Nucleotide metabolism				
DJ41_1568		2.7	0.02253	putative deoxyguanosinetriphosphate triphosphohydrolase
DJ41_2077		2.5	0.04054	cytidine and deoxycytidylate deaminase zinc binding region family protein
Peroxisome				
DJ41_405		2.7	0.03062	AMP binding enzyme family protein
PPAR signaling pathway				
DJ41_405		2.7	0.03062	AMP binding enzyme family protein
Purine metabolism				
DJ41_1568		2.7	0.02253	putative deoxyguanosinetriphosphate triphosphohydrolase
DJ41_2077		2.5	0.04054	cytidine and deoxycytidylate deaminase zinc binding region family protein
DJ41_881	*prs*	2.3	0.04751	ribose phosphate diphosphokinase family protein
Quorum sensing				

DJ41_405		2.7	0.03062	AMP binding enzyme family protein
Thermogenesis				
DJ41_405		2.7	0.03062	AMP binding enzyme family protein
Cell cycle - Caulobacter				
DJ41_1811		-2.5	0.01752	hypothetical protein
DNA replication				
DJ41_1811		-2.5	0.01752	hypothetical protein
Glycerolipid metabolism				
DJ41_2867	*lip*	2.5	0.03712	lactonizing lipase
Cysteine and methionine metabolism				
DJ41_2339		-2.3	0.03236	C 5 cytosine specific DNA methylase family protein
MicroRNAs in cancer				
DJ41_2339		-2.3	0.03236	C 5 cytosine specific DNA methylase family protein
Pentose phosphate pathway				
DJ41_881	*prs*	2.3	0.04751	ribose phosphate diphosphokinase family protein
Ubiquinone and other terpenoid-quinone biosynthesis				
DJ41_3060	*hppD*	2.3	0.04994	4 hydroxyphenylpyruvate dioxygenase
Pertussis				
DJ41_744		-2.1	0.03994	fimbrial family protein
Shigellosis				
DJ41_744		-2.1	0.03994	fimbrial family protein

Table S7. Differentially regulated genes in the Δ*DJ41_1407* mutant strain versus the wild-type strain ATCC 19606

Gene	log2 Ratio (W/Δ1407)	Annotation
DJ41_10	1.4	HD domain protein
DJ41_41	1.6	hypothetical protein
DJ41_84	1.1	short chain dehydrogenase family protein
DJ41_367	1.4	helix-turn-helix domain protein
DJ41_477	2.7	alpha/beta hydrolase fold family protein
DJ41_536	1.2	3-oxoacid CoA-transferase, A subunit
DJ41_542	19.6	hypothetical protein
DJ41_573	1.0	putative transcriptional regulatory protein
DJ41_878	1.5	putative ddrR
DJ41_967	1.3	hypothetical protein
DJ41_1407	20.0	PAS fold family protein
DJ41_1431	18.0	putative membrane protein
DJ41_1626	1.0	dihydroorotate dehydrogenase family protein
DJ41_1686	2.9	hypothetical protein
DJ41_1963	1.2	putative surface antigen
DJ41_1965	1.4	hypothetical protein
DJ41_2223	2.5	putative peptidoglycan domain-containing protein
DJ41_2264	1.1	hypothetical protein
DJ41_2358	1.3	prokaryotic cytochrome b561 family protein
DJ41_2868	1.2	proteobacterial lipase chaperone family protein
DJ41_3255	1.1	3-hydroxyisobutyrate dehydrogenase (mmsB)

DJ41_3283	22.6	hypothetical protein
DJ41_3439	3.2	hypothetical protein
DJ41_270	-1.3	hypothetical protein
DJ41_470	-18.3	bacterial regulatory s, tetR family protein
DJ41_559	-2.3	eamA-like transporter family protein
DJ41_564	-1.1	AAA domain family protein
DJ41_817	-20.1	XapX domain protein
DJ41_1032	-22.1	5S ribosomal RNA
DJ41_1035	-26.7	tRNA-Ile
DJ41_1182	-1.7	zonular occludens toxin family protein
DJ41_1224	-1.4	translation initiation factor IF-1 (infA)
DJ41_1408	-2.3	AAA domain family protein
DJ41_1452	-6.0	5S ribosomal RNA
DJ41_1497	-1.1	bacterial extracellular solute-binding s, 3 family protein
DJ41_1587	-1.1	sulfate ABC transporter, sulfate-binding family protein
DJ41_2314	-19.4	hypothetical protein
DJ41_2984	-1.1	tRNA-Ser
DJ41_3263	-19.0	asnC family protein
DJ41_3587	-17.8	hypothetical protein
DJ41_3653	-1.9	hypothetical protein
DJ41_3740	-1.1	5S ribosomal RNA
DJ41_3797	-18.2	hypothetical protein
DJ41_3804	-21.8	putative transcriptional regulator
